# Experience reorganizes content-specific memory traces in macaques

**DOI:** 10.1038/s41593-026-02357-2

**Published:** 2026-07-29

**Authors:** Saman Abbaspoor, Ayman Aljishi, Kari L. Hoffman

**Affiliations:** 1 Department of Psychology, Vanderbilt Vision Research Center, Vanderbilt Brain Institute, Vanderbilt University, Nashville, TN USA; 2 F. M. Kirby Neurobiology Center, Boston Children’s Hospital, Boston, MA USA; 3 Hansjoerg Wyss and Rosamund Stone Zander Translational Neuroscience Center, Boston Children’s Hospital, Boston, MA USA; 4 Department of Biomedical Engineering, Vanderbilt University, Nashville, TN USA; 5 Present Address: Department of Neurology, Harvard Medical School, Boston, MA USA

**Keywords:** Consolidation, Neural circuits, Hippocampus, Long-term memory, Replay

## Abstract

Memory formation relies on the reorganization of neural activity patterns during experience that persist in subsequent sleep. How these processes promote learning while preserving established memories remains unclear. Here we recorded neural ensemble activity from the hippocampal and associated regions in freely moving macaques as they recalled item sequences presented that day (‘new’), 1 day earlier (‘recent’) or more than 2 weeks earlier (‘old’). Cell assemblies biased for old sequences showed less drift, greater network connectivity and stronger sleep reactivation than new-biased assemblies. Pairs of old and recent assemblies formed persistent task-to-sleep coupling (‘metassemblies’), unlike new assembly pairs, and these assembly pairs showed longer interactions over time than the new pairs. In the hippocampus, the propensity for superficial and deep CA1 pyramidal cells to form integrated assemblies increased with memory age. These findings reveal rapid organization and stabilization of neural activity in the primate brain, suggesting potential mechanisms for balancing learning with memory linking and durability.

## Abstract (web summary)

In macaques, old memories, relative to new ones, are supported by more stable and interconnected cell assemblies. New-biased and old-biased assemblies reactivate during overnight sleep and can sometimes reactivate together, suggesting a mechanism for balancing new learning with memory linking and durability.

## Main

Memory formation and organization are dynamic, ongoing processes that allow the brain to encode, update and integrate multiple experiences over time. In rodents, accumulating evidence shows that recently formed memories are initially supported by selective activation of neuronal ensembles during learning and subsequently stabilized through their reactivation during offline states such as sleep^1–5^. Similar reactivation patterns have been observed in other species, including birds^6^, bats^7^, nonhuman primates^8,9^ and, more recently, in humans^10–12^. Although these findings suggest that some of the hippocampal circuit mechanisms are broadly similar across species, differences in ethological demands and behavioral repertoire may shape both the content of reactivated patterns—what types of experiences are prioritized for reactivation and retention—and the organization of temporally distinct experiences into an adaptive memory system. Much of the existing work has focused on stable representations of discrete, isolated and/or recent memories. Thus, it remains unclear how the brain manages to keep track of multiple distinct experiences over time and how it (re)organizes memories originating from different points in time^13–17^. This question is particularly relevant in primates, whose extended lifespans and cognitive flexibility require memory systems that support not only the preservation of individual experiences but also their integration into coherent, behaviorally adaptive representations. Although waking experience may recruit and support some of these processes^18–24^, offline states—and specifically sleep—are thought to play a crucial role in this reorganization^25,26^. In this study, we sought to address whether consistent activity patterns reflecting multiple spatiotemporal events (memoranda) are reactivated during sleep in macaques, and if so, how the patterns of activation and reactivation differ as a function of previous experience. Using high-density linear arrays to record wirelessly from hippocampal and extrahippocampal regions, we tracked experience-dependent changes in ensemble activity that could hold key insights into how the primate brain preserves older memories while incorporating new ones.

## Newly learned and remotely learned sequences can be decoded from synchronized spiking ensembles

**Fig. 1:**
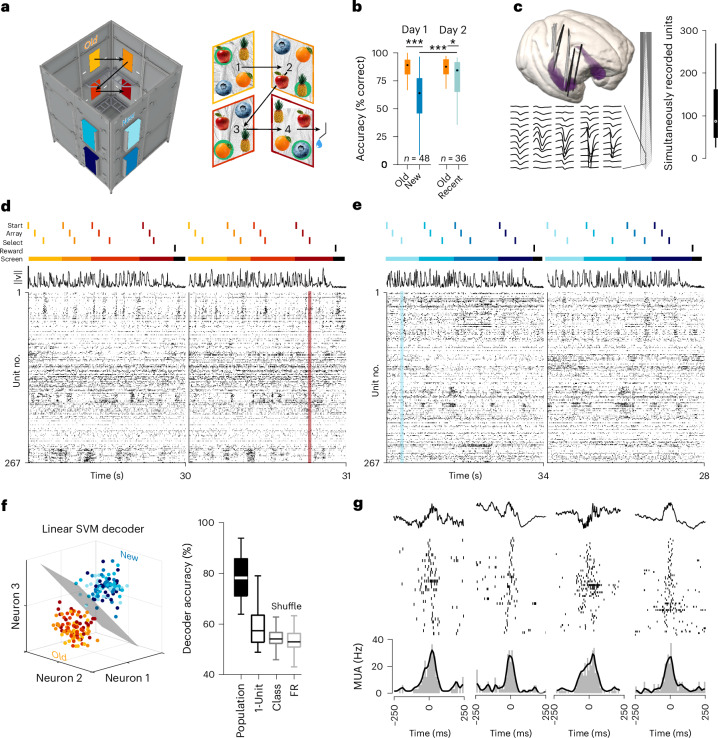
Experimental design and neural population analysis methods. **a**, Left: 3D rendering of the Treehouse 3D task environment indicating the opposite corners where old and new visual stimulus arrays are presented sequentially on touch screens. Right: trial schematic illustrating an example item-context sequence across four screens, with the correct target items circled in green. Item positions on the screens are randomized across screens and trials. Old and new item-context sequences (corners) are visited in alternating blocks^27^ (Methods). **b**, Target selection rates per sequence condition. The center circle shows the median accuracy across sets, the box shows the interquartile range and the whiskers show the most extreme nonoutlier values. Performance of new sequences improved between the first and second days of presentation (permutation test with FDR correction; *P* < 0.001), and performance of old sequences exceeded newly presented sequences (versus new on day 1; permutation test with FDR correction *P* < 0.001 versus recent on day 2; permutation test with FDR correction; *P* < 0.05), trial category label permutation tests). *P < 0.05, ***P < 0.001. **c**, Left: computed tomography (CT)–magnetic resonance imaging (MRI) reconstructed electrode trajectories for monkey 1 (black) and monkey 2 (gray) projected onto a macaque template brain^76^, with representative spike waveforms shown across adjacent recording channels from the high-density deep arrays. Right: isolated single-unit yields across sessions (monkey 1: *n* = 17 sessions; monkey 2: *n* = 18 sessions). The center circle shows the median number of isolated units across sessions, the box shows the interquartile range and the whiskers show the most extreme nonoutlier values. **d**, Top: timing of trial events across a sequence of four screens for two old trials. Middle: movement trace, shown as the vector norm of angular velocity. Bottom: raster plots of corresponding population spiking activity (time × cells), sorted according to the Rastermap method^77^; an example ‘population vector’ of activity highlighted in red. **e**, As in **d**, but for two new trials from the same session. **f**, Left: schematic of linear SVM decoding of trial type (old or new) based on population vector activity. Right: decoder accuracy of trial epoch identity classification for different SVM models (*n* = 26 population; 3,332 single units). The center line shows the median decoding accuracy across sessions, the box shows the interquartile range and the whiskers show the most extreme nonoutlier values. **g**, Single-unit population responses to SWRs from overnight recordings in macaques. Top: broadband local field potential (LFP) from CA1 aligned to ripple events. Single-unit responses are shown below, centered on the ripple event. Bottom: response histogram averaged across units. Modulation to SWRs in these recordings has been quantified previously^47^.

**Extended Data Fig. 1:**
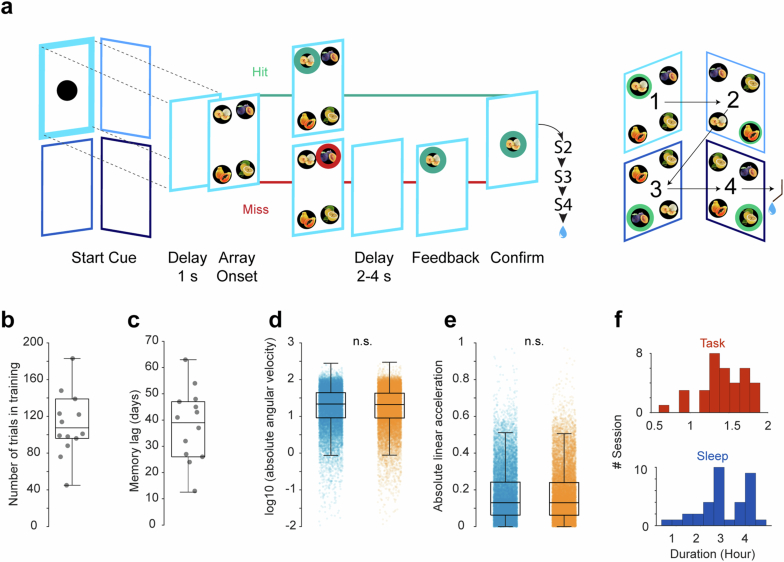
Sequential item-in-context association task. **(a)** Left: Trial sequence for the first screen in the task sequence. The bold outline represents the active screen; the green ring indicates the correct target, and the red ring indicates an incorrect distractor. After completing this procedure on one screen, the trial proceeds to the next screen in the sequence finishing with reward, which followed every 4^th^ screen completion. Right: Four objects and example positions on the screen for one trial. Green rings indicate the associated target objects assigned to each screen. The assignment of objects to the four screen positions in the 2 × 2 array is randomized across screens and trials, preventing prediction of object location, and the background image is simplified for illustration purposes. **(b)** Distribution of the number of training trials prior to the first day of recall for the old set (n = 13 sets). **(c)** Distribution of memory lag. Number of intervening days between the end of initial exposure to a stimulus set during learning and its return as the ‘Old’ category (n = 13 sets). **(d)** Distribution of the angular velocity values (degrees per second) during trial epochs (n = 17286 timepoints, n.s., two-sided permutation test). **(e)** Distribution of the linear acceleration values (m^2^/s) during trial epochs (n = 17286 timepoints, n.s., two-sided permutation test). New set: Blue, Old set: Orange. **(f)** Distribution of wireless recording durations during task performance (red, top) and sleep (blue, bottom). The box plots in b-e show the median and interquartile range, and the whiskers show the most extreme non-outlier value.

**Extended Data Fig. 2:**
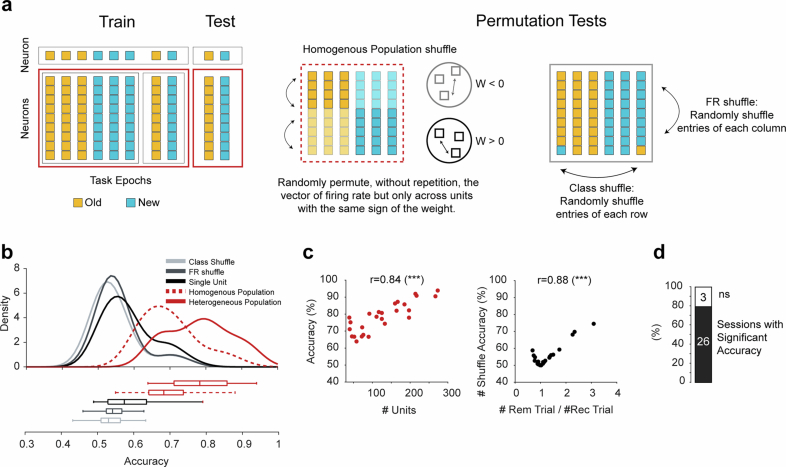
Decoding behavioral context from population spiking activity. **(a)** Schematic representation of the linear support vector machine (SVM) training and testing approach. Left: Main (heterogeneous) population decoding and single-unit decoding. Middle: Homogeneous population decoding. Right: Class and firing rate shuffling tests for significance (see Methods and Ref. ^80^ for details). **(b)** Kernel density distributions of SVM cross-validation accuracies for different design matrices (n = 26 sessions). The box plots show the median decoding accuracy and interquartile range, and the whiskers show the most extreme non-outlier value. **(c)** Top: Correlation between the number of units included in population decoding and SVM performance accuracy. Bottom: Correlation between trial number balance across conditions and the accuracy of the shuffled design matrix. **(d)** Proportion of sessions with significant decoder accuracy.

**Extended Data Fig. 3:**
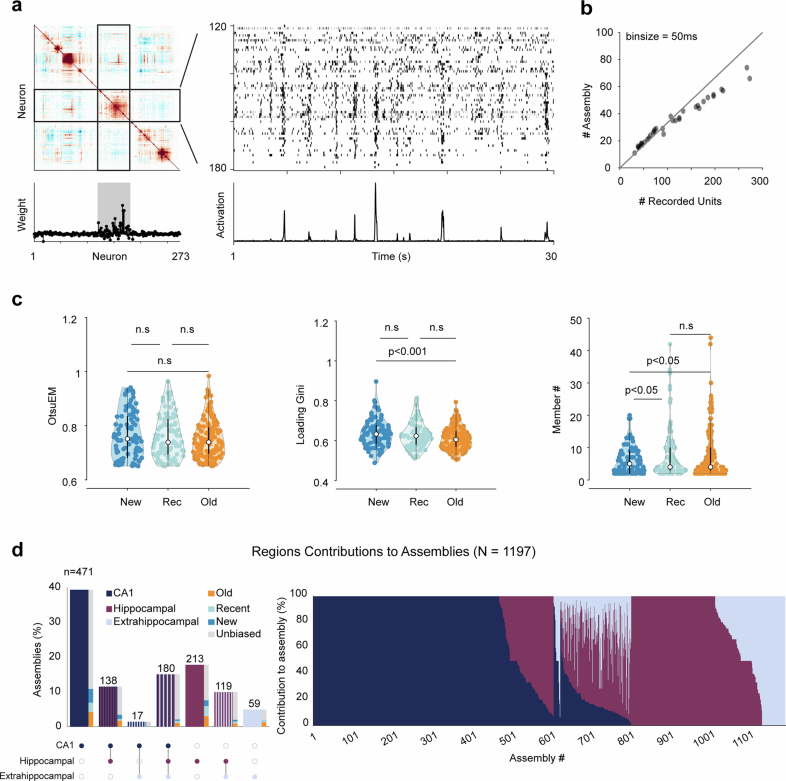
Cell assembly detection, quality control, and region composition. **(a)** Top-left: Correlation matrix (r) showing pairwise correlations between units in an example session. Top-right: Firing rate traces of a subset of neurons exhibiting highly correlated activity. Bottom-left: Example cell assembly pattern (1 of 66 detected during the session), with significant cell assembly members highlighted in gray. Bottom-right: Activation strength trace of the highlighted cell assembly and the corresponding firing rate raster. **(b)** Relationship between the number of recorded units in a session and the number of detected assemblies using the PCA/ICA method. **(c)** Left to right: Distributions of OtsuEM values, loading Gini coefficients, number of member neurons per assembly (n = 94, 92, 147 assemblies for New, Recent and Old, respectively). Statistical tests are based on pairwise differences from two-sided permutation tests with 10000 permutations and FDR correction. The box on violin plots shows the median and the interquartile range, and the whiskers extend to the most extreme data points within 1.5 × the interquartile range from the lower and upper quartiles. **(d)** (left) Percentage of assemblies that are comprised of cells from the indicated locations (see Methods). The strip on the right side of each bar plot further indicates the proportion of those assemblies belonging to New/Recent/Old/No-bias groups. (right) For each assembly, the percent contribution of each location is shown, sorted in order of the bars on the left plot.

We used high-density linear arrays to simultaneously record single-unit activity from neuronal ensembles in the hippocampus and connected extrahippocampal regions of two freely moving macaques (range: 31–273 units per session; *n* = 35 sessions; Fig. 1c–e and Extended Data Fig. 1b–d). Task recordings took place in ‘Treehouse’, a touch-screen-enabled three-dimensional (3D) enclosure, followed by sleep recordings taken overnight in their housing room (Fig. 1a,g and Extended Data Fig. 1a,f). ‘Day 1’ Treehouse sessions consisted of two types of repeated Z-shaped trajectories: a new item-context sequence with four items spanning the four touch screens in one corner and an ‘old’ sequence presented in the opposite corner that had been learned in that corner, on average, 6 weeks earlier with no intervening exposure (Fig. 1a, Methods and Extended Data Fig. 1b,c). This allowed simultaneous recording during the performance of two sequences that differed in their previous exposure but with the corner location of the old or new sequence types counterbalanced across sets (Fig. 1c–e and Extended Data Fig. 2). Individual learning results in this task have been reported previously^27^. Accuracy was higher for old sets than for new sets, indicating long-term memory savings (Fig. 1b; *P* < 0.001; permutation test with false discovery rate (FDR) correction). ‘Day 2’ sessions occurred the following day and used the same stimulus sets and assigned locations as day 1, with the new set now termed ‘recent’. Day 2 performance on recent sets improved (*P* < 0.001; permutation test with FDR correction) while remaining below the level of old sets, suggesting overnight retention of the newly learned sequences and a residual benefit of long-term, remote memory for the old sequences. The recorded neural activity varied with task demands (Extended Data Fig. 3a–d). Thus, to determine whether the recorded neural activity could contribute to selective memory for these sequences, we trained a linear support vector machine (SVM) decoder to classify trial identity (old versus new or recent) based on single-unit or ensemble activity (Fig. 1f and Extended Data Fig. 2). Decoding based on ensemble activity consistently outperformed decoding that used single-unit activity and also surpassed shuffling controls (Fig. 1f and Extended Data Fig. 2). Movement-related variables during the task, including angular velocity and linear acceleration, did not differ between the new and old sequences performed in their respective corners and are therefore unlikely to account for the observed decoding performance (Extended Data Fig. 1d,e). These results indicate that the pattern of spiking activity in the recorded ensemble generally varied as a function of trial identity.

**Fig. 2:**
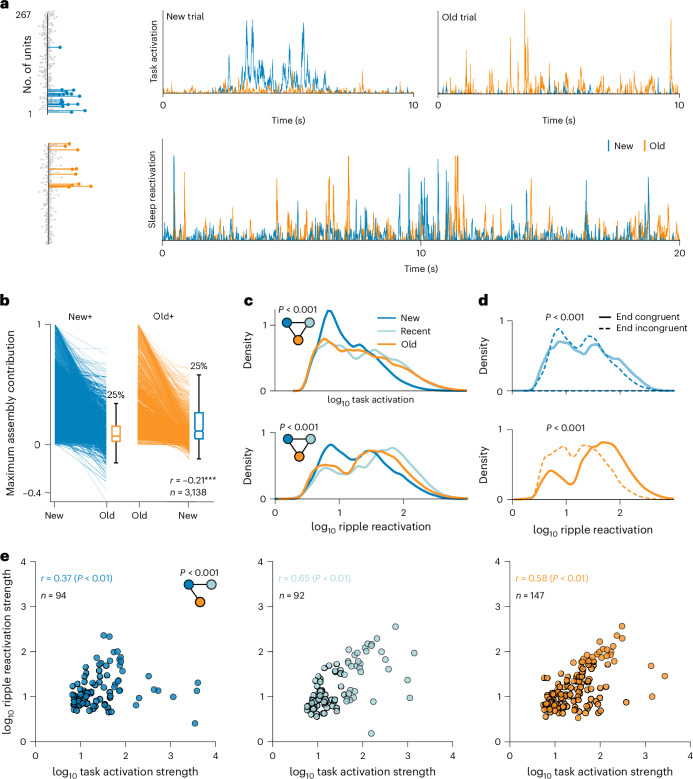
Activation of task-selective cell assemblies and their reactivation during sleep SWRs. **a**, Task-selective cell assemblies. Left: examples of cell assemblies from the same session, with member weights colored according to biased activation for old (orange) or new (blue) conditions. Top: activation strengths of both assemblies (blue and orange, respectively) presented for a new-sequence trial (left) and an old-sequence trial (right) demonstrating biased activation by trial. Bottom: sleep reactivation strengths of the two example cell assemblies. **b**, Polarization of single-unit assembly contributions (*n* = 3,138 single units). The maximum assembly contribution of each single unit was calculated for each new/old category. Shown on the left are those with greater new contributions (‘new+’, blue) and on the right are those with greater old contributions (‘old+’, orange). Box plots show the weights for the nonpreferred category obtained from cells with weights in the top quartile of the preferred category, suggesting an inverse relationship. (***P < 0.001; Spearman correlation on pooled groups). The center line shows the median, the box shows the interquartile range and the whiskers show the most extreme nonoutlier values. **c**, Distributions of activation during the task (top) and sleep reactivation during ripples (bottom). Strengths were pooled across assemblies of the respective categories (new: blue; recent: light blue; orange: old). Inset: *P* < 0.001 pairwise distribution differences indicated by black lines connecting color-coded circles; two-sided permutation test. **d**, Distributions of reactivation strength for new (top) and old (bottom) assemblies, shown separately based on the old/new context of the last trial block. ‘End congruent’ refers to cases in which the context of the last trial block matches the activation bias of the cell assembly. ‘End incongruent’ refers to cases in which the final context and activation bias are mismatched (***P < 0.001; two-sided pairwise permutation test). **e**, Scatterplots of mean sleep ripple reactivation strength per assembly as a function of mean task activation strength. Left: new-sequence biased assemblies; middle: recent-sequence assemblies; right: old-sequence assemblies. Inset, left: the black line connecting dots indicates significant differences in activation–reactivation correlation between cell assembly categories, as represented by dot color (*P* < 0.01; Spearman correlation with permutation. *P* < 0.001; Fisher’s *z*-test with FDR correction).

**Extended Data Fig. 4:**
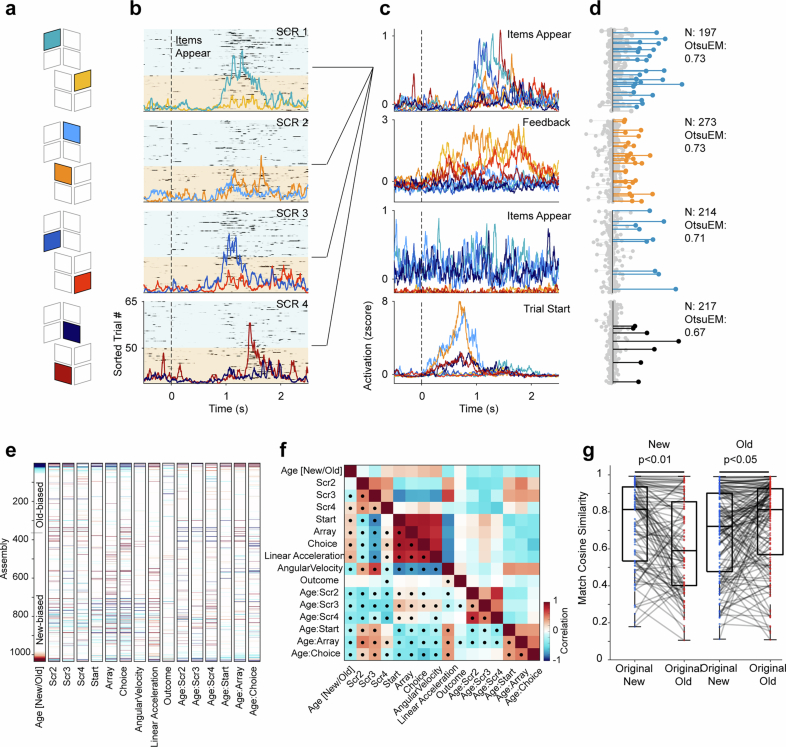
Context-biased cell assembly activation during task. **(a)** Schematic of screen locations in the 3D task environment “Treehouse.” **(b)** Activation strength across trials and screens for an example assembly, along with its average activation per screen. Trials are sorted by context for clarity (New: blue; Old: orange). Average normalized activation strengths are color-coded by the screen location during each trial. **(c)** Examples of context-biased and/or task-selective activations during different task epochs. Each plot shows the average activation trace of a different cell assembly across screen locations within a given context. Traces are color-coded by screen location. The top plot is the assembly example shown in (a). **(d)** Assembly templates (loadings) corresponding to the assemblies shown in (c). Significant weights are color-coded by bias (New: blue; Old: orange; Nonbiased: black). N indicates the number of neurons included in the PCA/ICA analysis. OtsuEM represents the effectiveness measure of Otsu thresholding. Only high-quality assemblies with an OtsuEM > 0.65 were included in the biased cell assembly analysis. **(e)** Cell assembly activations modulated by diverse task variables. Distribution of the beta coefficient weights for the generalized linear model. Nonsignificant beta coefficients (p > 0.05) were set to 0 for the visualization. Beta coefficients are sorted based on the age column. **(f)** Pairwise correlations of the beta coefficients vectors (in g.) showing the task-related covariates across the population of cell assembly activations. Dots indicate significant correlation values (p < 0.05, permutation test with FDR correction). **(g)** After restricting the population activity during one sequence type (that is New or Old), independent sets of cell assemblies were detected for each type. Shown on the left are the cosine similarities between each New-restricted assembly and its best match from the original “new-biased” assemblies and from the original “old-biased” assemblies (n = 94 assemblies). The right side depicts the same best-match procedure, but for the Old-restricted assemblies (n = 147 assemblies). The matches to the congruent assemblies (restricted New to the original New-biased and restricted Old to the original Old-biased) were closer than to those of the incongruent group (Two-sided Wilcoxon rank-sum test; New: p < 0.01; Old: p < 0.05). The center line shows the median similarity, the box shows the interquartile range, and the whiskers show the most extreme non-outlier values.

We used component analysis (principal component analysis (PCA) or independent component analysis (ICA)^28–30^; Methods) to identify distinct ensembles of coactive neurons (Fig. 2a and Extended Data Fig. 4) as an operational definition of ‘cell assemblies’. The composition of assemblies by members’ location is shown in Extended Data Fig. 3d. We then fitted a generalized linear model (GLM) to each detected assembly to assess how well activation strength could be predicted by specific trial elements, such as sequence position, behavioral outcomes (for example, trial success) and movement parameters. This approach also allowed us to examine which factors tended to covary across assemblies (Extended Data Fig. 4e,f). To determine whether these cell assemblies reappear during postlearning sleep and whether offline processing differs between assemblies biased toward new versus old information, we grouped cell assemblies into old, new/recent or nonselective categories based on their activation likelihood between the two stimulus sequences. Notably, these categories of assemblies did not differ in quality metrics (Otsu’s effectiveness metric (OtsuEM)^31,32^) or in the firing rates of their member neurons (Extended Data Figs. 4c and 8a), indicating that key features relevant for detecting and defining cell assemblies were comparable across groups. Old-biased and recent-biased assemblies were, however, moderately larger (had more members on average) than new-biased assemblies (Extended Data Fig. 4c). We assessed whether the strength of contributions from a cell to one assembly might determine its contributions to other assemblies. We found that cells acting as strong contributors to the ‘best’ assembly of a category (old or new) predicted much weaker strength to the best assembly of the other group (an inverse correlation of strength), indicating a polarization of member responses by category (Fig. 2b). Finally, to check the robustness of the association to each sequence type, we ran a separate analysis in which assemblies were recalculated when activity windows were limited to the task sequences of one type (one set for old trials and a separate set for new trials). For each new set of assemblies, we identified the original assembly with the greatest cosine similarity to the old-biased group and to the most similar assembly from the new-biased group. We found that congruent matches (for example, new to new) were stronger, suggesting consistent activity covariance within each corner independently (Extended Data Fig. 4g; Wilcoxon rank-sum test; new: *P* < 0.01; old: *P* < 0.05).

## Experience biases sleep reactivation of task-related cell assemblies

**Extended Data Fig. 5:**
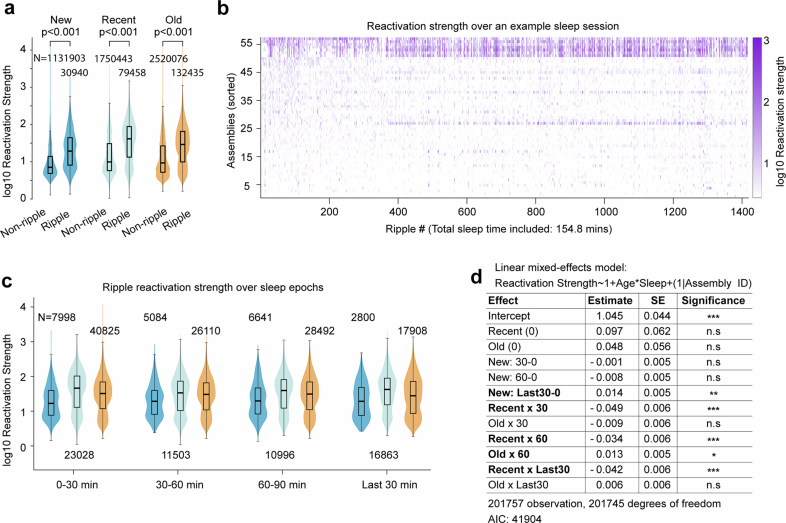
Cell assembly dynamics during sleep. **(a)** Reactivation rate during sharp-wave ripples and excluding ripple periods during sleep, organized by assembly group. p < 0.001, two-sided pairwise permutation test. **(b)** Reactivation strengths of assemblies during an example sleep recording, sorted by initial reactivation strength (N = 57 assemblies). **(c)** Ripple reactivation of each assembly group, restricted to 30-minute epochs from the first 1.5 hours of sleep and the last 30 minutes (N=number of reactivation events). **(d)** Linear mixed effects model of the influence of assembly type (Age), Sleep bin, and their interaction. The box on violin plots in a and c shows the median and the interquartile range, and the whiskers extend to the most extreme data points within 1.5 × the interquartile range from the lower and upper quartiles.

We tracked assembly activation during task and reactivation in sleep and found that all categories of cell assemblies reactivated in sleep, predominantly during ripples (Fig. 2a and Extended Data Fig. 5a). Old-biased and recent-biased assemblies showed greater and more frequent activation and reactivation than new-biased assemblies (Fig. 2c; *P* < 0.001; permutation test with FDR correction). All three task groups showed linear correlation between task activation and sleep ripple reactivation (Fig. 2e); however, this correlation was stronger for the old and recent cell assemblies compared to the new assemblies, with no difference between old and recent (Fig. 2e; *P* < 0.01; Spearman correlation with permutation test. *P* < 0.001; Fisher’s *z*-test with FDR correction). In addition to the preservation of relative assembly strength into sleep, both new and old assemblies showed a reactivation recency bias; they were more strongly reactivated when their respective sequence was the last one experienced before sleep (Fig. 2d). In contrast, the group difference in reactivation strength during different periods of sleep was relatively constant, even as specific assemblies show individual time courses of reactivation (Extended Data Fig. 5b–d). Overall, this recapitulates findings from rodent studies^21,33,34^ and reinforces the idea that both new and old assemblies are well formed, yet their reactivation in sleep is subject to task-related influence.

## Assemblies link and stabilize over time

**Fig. 3:**
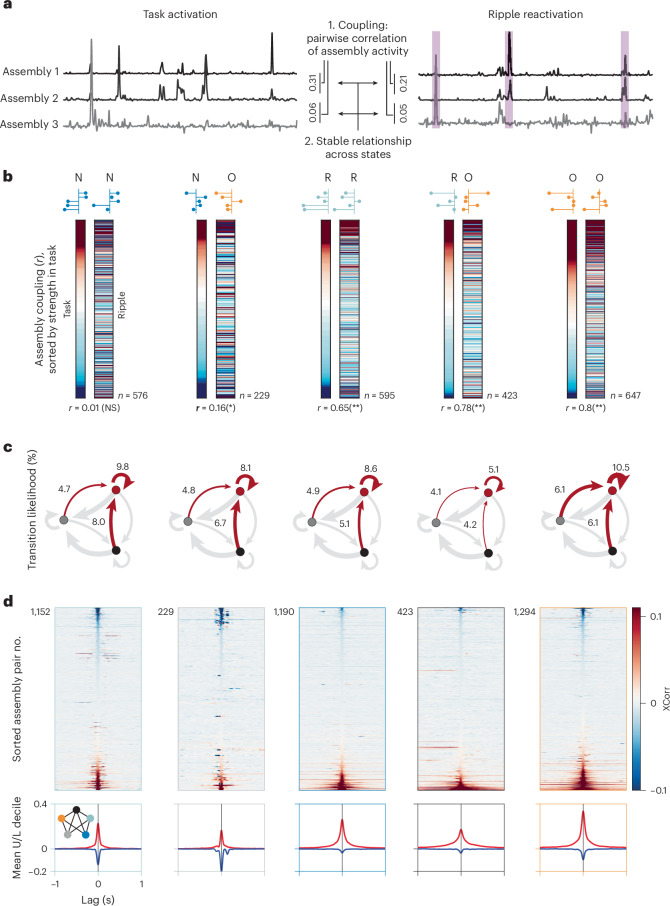
Changes in assembly coupling with memory age. **a**, Assembly coupling 1 was calculated as the pairwise correlation of activation strengths for each assembly pair during the task and again during ripples. Shown are the coupling values obtained from three assemblies (two pairs: 1 and 2, and 2 and 3) during the task and separately during ripples. Across all pairs within a given age category, the consistency of coupling 2 between task and sleep was measured using a Pearson correlation. Purple shading indicates time windows corresponding to detected ripples. **b**, Color-coded plots of coupling strength (coactivation or coreactivation) between pairs of cell assemblies across different memory categories during task events and sleep ripple events. Pairs were sorted based on task activation coupling strength. The consistency of coupling across task and ripple epochs was quantified as the Pearson’s correlation, shown below each plot (*P < 0.05, **P < 0.01). **c**, Average Markov state transition likelihoods between assemblies. Transition states are activation (red), baseline (gray) and suppression (black). Only transitions to the active state are shown, with arrow thickness indicating greater transition probabilities. Note that the other transitions are depicted schematically and are identical across plots. See Methods and Extended Data Fig. 6 for the full transition table. **d**, Top: assembly cross-correlograms for pairs of each memory type, sorted by strength at 0 lag. Bottom: averages of the first (upper, red) and last (lower, blue) deciles of the sorted responses. The inset shows differences in the full-width at half-maximum lags of these groups (*P* < 0.01; permutation test with FDR correction), organized in descending order of the longest lags. Both magnitude and longevity of coactivations vary across groups and show similar patterns to the Markov state transitions. NS, nonsignificant; U/L upper, lower.

**Extended Data Fig. 6:**
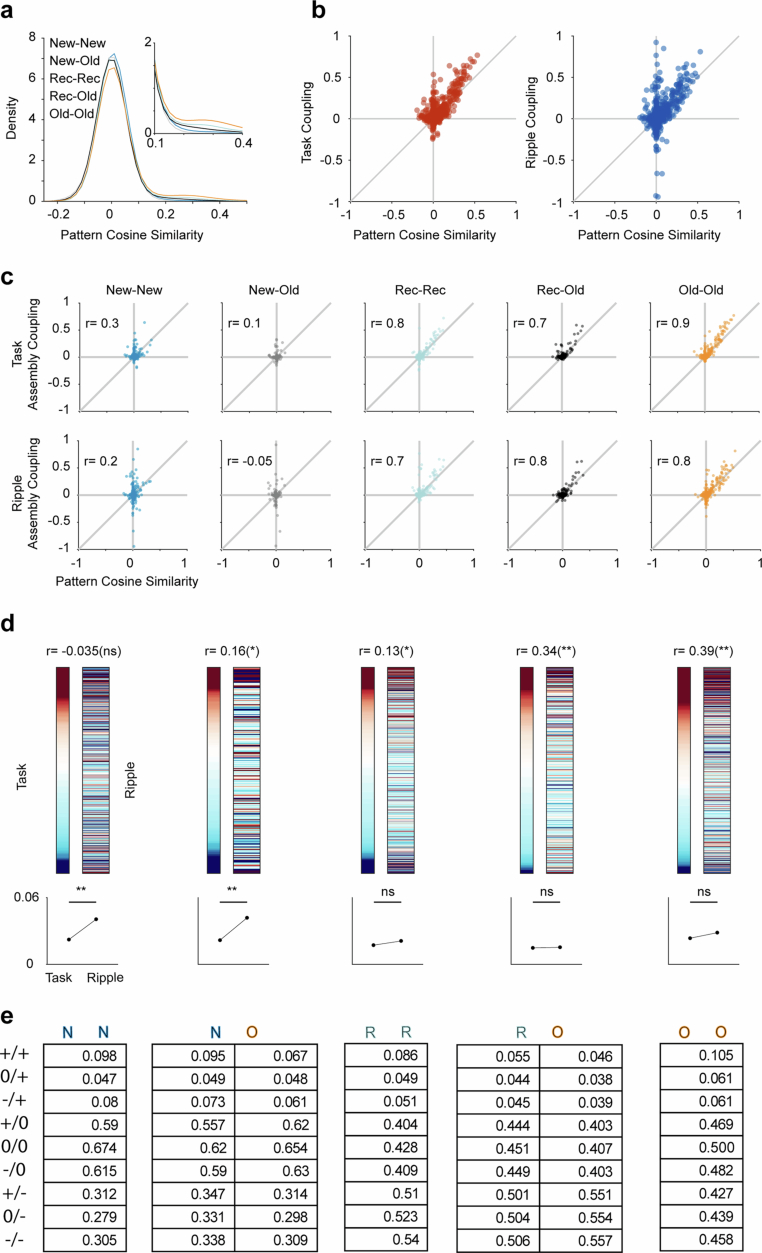
Cell assembly coupling. **(a)** Distribution of pairwise pattern cosine similarity between different categories of cell assemblies. **(b)** Relationship between pattern cosine similarity and cell assembly coupling across various brain-behavioral states. **(c)** Assembly coupling during task (top) and sleep ripple (bottom) as a function of pattern cosine similarity, separated by cell assembly category. **(d)** Same as Fig. 3 but calculated exclusively for pairs of cell assemblies with cosine similarity < 0.15. **(e)** Shown for each assembly pair group is the average transition probabilities from/to the activated (+), Baseline (0), and suppressed (-) states.

Emerging evidence indicates that, at the neuronal level, the hippocampus may support flexible, higher-order linking of smaller neuronal assemblies into larger integrated networks. Such linking could facilitate rapid learning and efficient encoding of complex experiences^35^. Offline processes during rest or sleep may play a critical role in enabling such associations. If two cell assemblies are linked, the correlation between their activation strength vectors should increase; if this coupling is stable, the correlation should remain consistent across time and behavioral states. To assess this possibility, we computed the correlation between pairs of assembly activations, during the task and separately during sleep ripples (assembly coupling; Fig. 3a,b and Extended Data Fig. 6). Pairs of assemblies with recent and old memory biases exhibited greater stability in their correlations across task and sleep—evident by the high ‘second-order’ correlations between task and sleep—whereas coupling among new–new and new–old pairs showed greater reorganization across task and sleep groups (Fig. 3b). We observed the same pattern of results even after excluding assembly pairs with high cosine similarity (Extended Data Fig. 6d), confirming that the observed changes in coupling were not driven by redundant assembly membership (composition) but rather reflect genuine experience-dependent reorganization of inter-assembly dynamics. Changes in higher-order cell assembly coupling could stem from the cofiring changes of constituent neurons. We calculated cofiring as the pairwise correlation between neuron firing rates, comparing task versus sleep cofiring within ripples to assess stability across states (Extended Data Fig. 9). Average cofiring was greater for members of the old and recent assemblies than for those of the new assemblies (*P* < 0.001; permutation test with FDR correction).

We next addressed the temporal relationship between assemblies in two ways. First, to characterize discrete modes of expression, we modeled each assembly time series as a three-state Markov process: active, baseline and suppressed. We then calculated lagged state transitions across assemblies for each possible pair group. Because we are especially interested in the likelihood of transitioning to an activated state, we show the three types of transitions to active state for each of the cell pair groups in Fig. 3c (see also Extended Data Fig. 6e for the full transition matrix). Considering the three states of a given assembly, the relative likelihood of entering an active state from the active state of another assembly was greatest for the old–old pairs (10.5%) and was generally higher during same-group transitions (new–new: 9.8%; recent–recent: 8.6%). In contrast, the group with the greatest likelihood of transitioning from an inactive assembly to an active assembly was the new–new group (8%). Do these discrete transitions translate to time-lagged activation between assembly pairs?

To address this question, we ran pairwise cross-correlations for members of each group (Fig. 3d) and isolated the average responses of the largest and smallest 10% of correlations at 0 lag, revealing apparent differences in time-extended pairwise activation. To quantify the lag, we calculated the full-width at half-maximum values of the top 10% of absolute correlations for each group and found differences across all but the new–new and new–old groups, which were the weakest, and the old–old and recent–recent groups. The ‘oldest’ pairings showed the most time-extended correlations, with some pairs extending as long as 500 ms or longer at half-maximum.

These results suggest that sleep facilitates the emergence of higher-order interactions among assemblies, which stabilize with experience. The formation of such metassemblies—hierarchically organized groups of assemblies with varying degrees of association—may support flexible reorganization of assemblies for encoding new memories while preserving core ‘elemental’ assemblies linked to older experiences. Selective linking of assembly subsets and their elaboration in time could further enable the integration and temporal chaining of new memories into pre-existing mnemonic frameworks.

## Network mechanisms of cell assembly dynamics

**Fig. 4:**
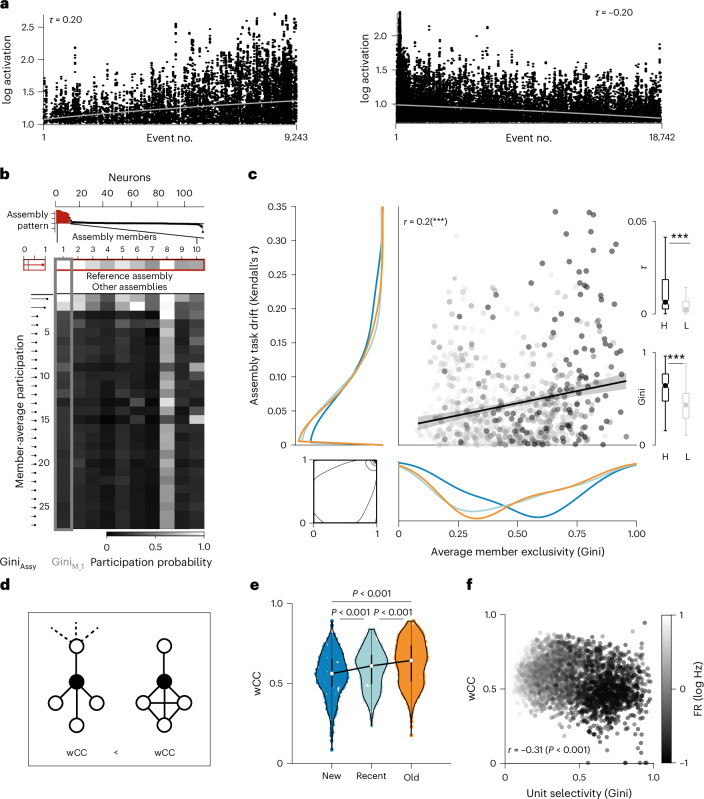
Network properties of assembly reorganization. **a**, Examples of drifting cell assemblies with positive and negative drift in activation strength across the task, indicated by white lines representing the Theil–Sen estimator. **b**, Schematic representation of assembly exclusivity and member selectivity quantification. Top: a reference assembly pattern expanded to show the participation of its members (red box). Below: participation probability matrix depicting the participation probability of each of the reference assembly members (columns) for a given alternate assembly (rows) for all detected cell assemblies within a session. Left: average participation probability of the target members during each detected cell assembly in the session. Higher average member exclusivity of a reference cell assembly is indicated by lower average member participation probabilities across all but the reference cell assembly, measured as the Gini_Assy_ coefficient. A selectivity index was calculated for each member (unit) based on the distribution of participation probabilities across all detected cell assemblies (Gini_M_no._) (see Methods for details). **c**, Scatterplot showing the relationship between assembly drift during the task (Kendallʼs *τ*) and the average exclusivity of assembly members (Gini coefficient), with marginal distributions separated by memory category (*n* = 922 assemblies). Points are grayscale coded based on absolute changes in mean correlations from the first 25% to the last 25% of cell assembly activation events in the task (see Methods for details). The line represents the best linear fit with 95% confidence intervals, indicating a significant correlation from a permutation test (*P* < 0.001). The best-fitted copula model (Gumbel, *θ* = 1.20) is used to illustrate extreme joint positive dependencies, suggesting that the relationship is pulled by the most extreme points (exclusive, drifting assemblies). Right: distributions of Kendallʼs *τ* (top) and average member task participation Gini (bottom) for cell assemblies with the highest 25% (black) and lowest 25% (gray) changes in absolute correlation (***P < 0.001; two-sided permutation test). The center line shows the median number of *τ* (top) and Gini (bottom), the box shows the interquartile range and the whiskers show the most extreme nonoutlier values. **d**, Schematic example illustrating two neurons with identical numbers of connections but different wCCs, based on the connection strength among their neighboring neurons. The wCC incorporates edge weights to quantify the density and cohesiveness of local connections. **e**, Violin plots depicting the distribution of wCC among assembly members across different categories (*n* = 568, 745 and 1,179 cells of new, recent and old groups, respectively). *P* < 0.001; pairwise differences from two-sided permutation tests with 10,000 permutations and FDR correction. **f**, Scatterplot of wCC as a function of selectivity in which each dot represents a neuron, grayscale-coded based on the log_10_ firing rate of that neuron (*n* = 3,174). Spearman correlation (*r*); *P* < 0.001; two-sided permutation correlation test.

**Extended Data Fig. 7:**
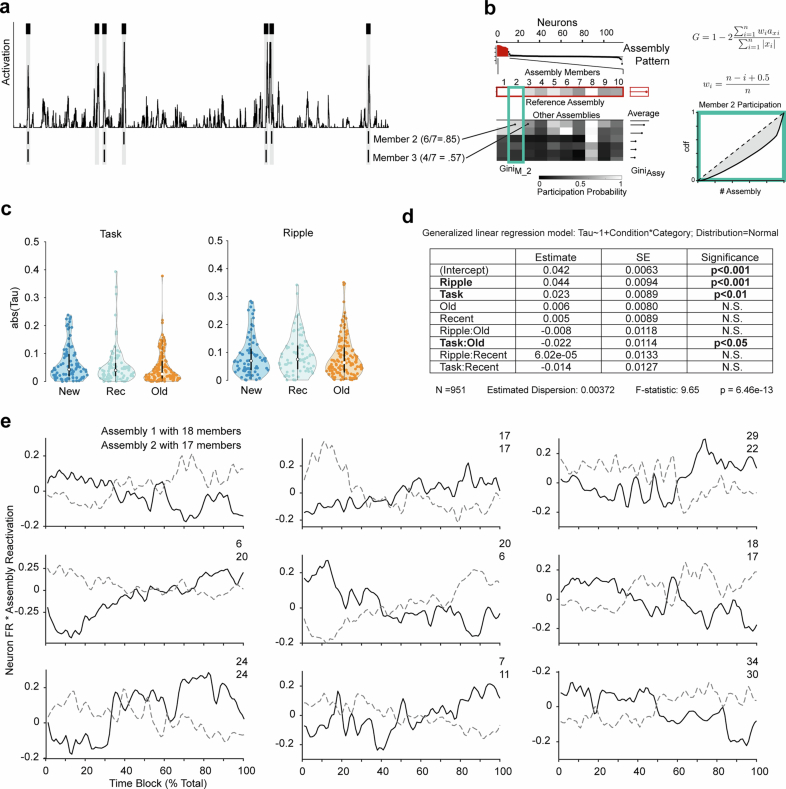
Modulation of cell assembly drift. **a)** Activation traces for an assembly above the spiking activity of two member neurons. The participation probability of a neuron in a specific cell assembly was calculated as the number of activation events where the neuron fired at least one spike, divided by the total number of detected activation events. **(b)** Participation probability matrix (abbreviated from Fig. 4) for members of a reference cell assembly (red) during activation events of all detected cell assemblies within a session. The right plot shows the average participation probability of the target members during each detected cell assembly in the session. Higher member exclusivity of a reference cell assembly is indicated by lower average member participation probabilities across all but the reference cell assembly (Gini_Assy’). A selectivity index was calculated for each member based on the distribution of participation probabilities across all detected cell assemblies. The selectivity index was determined as the Gini coefficient of the participation probabilities, G in equation to the right (see Methods section for details). The box on the right illustrates the principle of measuring the equality of contributions across assemblies as the curve away from the diagonal in the cdf plot (the Gini index). **(c)** Distribution of absolute Kendall’s tau values for different categories of cell assemblies during task (n = 92, 92, and 146 assemblies for New, Recent and Old, respectively) and sleep ripple (n = 75, 77, 134 assemblies for New, Recent and Old, respectively). For each condition, only assemblies with at least 25 activations in that condition were included in the analysis. The box on violin plots shows the median of the absolute Kendall’s tau across assmeblies, and the interquartile range, and the whiskers extend to the most extreme data points within 1.5 × the interquartile range from the lower and upper quartiles. **(d)** Generalized linear model (GLM) predicting drift (Kendall’s tau) using the cell assembly features: state (Ripple, Task) and sequence age type (New, Old). **(e)** Each panel shows the correlation between the spiking activity of an example single neuron and the reactivation strength of two selected assemblies, computed across 100 equal-length time blocks during the sleep session. The number of members in those assemblies is shown on the right of each plot, and relative elapsed time is shown. Note that even as the coupling between single neuron spiking and assembly reactivation gradually drops (negative drift) in one of the pairs, the strength of response in the other assembly can remain strong or increase.

Changes from task activation to sleep reactivation, cell assembly coupling and pairwise neuronal cofiring indicate that cell assemblies undergo reorganization based on experience. Yet most methods for assessing assembly reactivation or replay assume a stationary activation ‘template’ at least within the reference session^4,36^. In particular, the approach involving PCA and/or ICA defines assemblies statically and assumes temporal stationarity within the reference session, failing to capture changes in the structure of neuronal correlations over time. Recent findings suggest that neural ensembles dynamically reorganize to update memories and track ongoing experience^37–42^. In our study, if cell assemblies reorganize through changes in the firing rates of participating neurons or in the structure of their correlations over time, we would expect to observe corresponding trends in their activation during the task or reactivation during sleep. We identified a subset of ‘drifting assemblies’ with activation strength that changed gradually across task and sleep sessions (Fig. 4a and Extended Data Fig. 7). Assembly drift was seen across assembly type and task and sleep epochs but was greater during ripples (Extended Data Fig. 7; GLM *β* = 0.044; s.e. = 0.009; *t*(942) = 4.67; *P* < 0.001) and task (*β* = 0.024; s.e. = 0.009; *t*(942) = 2.65; *P* = 0.008) relative to the reference sleep nonripple epochs, and showed an interaction whereby old assemblies exhibited less task drift (*β* = −0.023; s.e. = 0.011; *P* < 0.05; Extended Data Fig. 7). To determine whether the group differences in drift rate were due to differences in assembly size, we compared assembly size to drift rates for each group but failed to find a correlation (Spearman correlation: new *r* = −0.092 and *P* < 0.378; recent *r* = −0.105 and *P* = 0.321; old *r* = −0.140 and *P* = 0.091). Examples of single-unit spiking that altered coupling with one member assembly without concomitant changes to another member assembly are shown in Extended Data Fig. 7e.

Having observed assembly drift as a regular phenomenon, we evaluated whether drift might differ as a function of specific assembly characteristics. Constituent neurons of assemblies can switch between assemblies, thereby changing their participation dynamics in the activation of a specific assembly^43^. A cell participating in many assemblies may be less able to maintain its strength among members of any one assembly as a result of mechanisms such as heterosynaptic plasticity^44,45^. Accordingly, one might predict that assemblies with members more exclusive to that assembly could provide greater stability (less drift) than those with highly promiscuous members participating in competing activity patterns. To investigate whether there is a relationship between the membership exclusivity of a cell assembly and its future drift, we computed the average participation Gini coefficient of each member, which is a measure of selectivity (see Fig. 4b, Extended Data Fig. 7 and Methods for details), and correlated it with our measure of drift. New assemblies had higher average Gini coefficients, suggesting increased selectivity in their members (Fig. 4c; pairwise permutation tests; 10,000 permutations: new versus recent, *P* = 0.0026 and new versus old, *P* = 6 × 10^−4^; old versus recent, *P* = 0.804). Furthermore, and in contrast to the predicted drift rates, assemblies with more exclusive members showed greater drift than those with more promiscuous members (Fig. 4c; *r* = 0.2; *P* < 0.001; Pearson’s correlation with permutation test).

These results align with our findings on cell assembly coupling, in which both old and recent cell assemblies displayed greater inter-assembly correlation and inter-member cofiring. We also measured changes in pairwise cofiring across the initial and final 25% of activation events for each cell assembly and examined their relation to both membership exclusivity and drift. Cell assemblies exhibiting the largest drift and membership exclusivity also showed the greatest changes in cofiring (Fig. 4c). One interpretation of this result is that assemblies that are initially isolated may tend to drift toward greater integration within a larger, overlapping network. Observations in rodents indicate that drift results from alterations in higher-order network properties; richly connected neurons are less likely to be dropped from an assembly^39,46^. Here the relation between experience (age of the assembly) and network connectedness was unclear. Therefore, we measured the clustering coefficient for members of cell assemblies to see if their constituents differed as a function of age category (Fig. 4d–f). The clustering coefficient reflects the extent to which the neighbors of a node form interconnected clusters, indicating local network density and structure (Fig. 4d). The members of each assembly contributed their weighted clustering coefficient (wCC) to the corresponding age group, resulting in larger wCCs with increasing age (new < recent < old), suggesting that with extended experience, assemblies exhibit increased interconnectedness and local cohesion, which may facilitate more stable and resilient network structures essential for long-term memory processing (Fig. 4e). Additionally, we observed a relationship between wCC and cell selectivity in specific assembly activations, further influenced by neuronal firing rates (Fig. 4f).

## Physiological features of assembly member neurons

**Extended Data Fig. 8:**
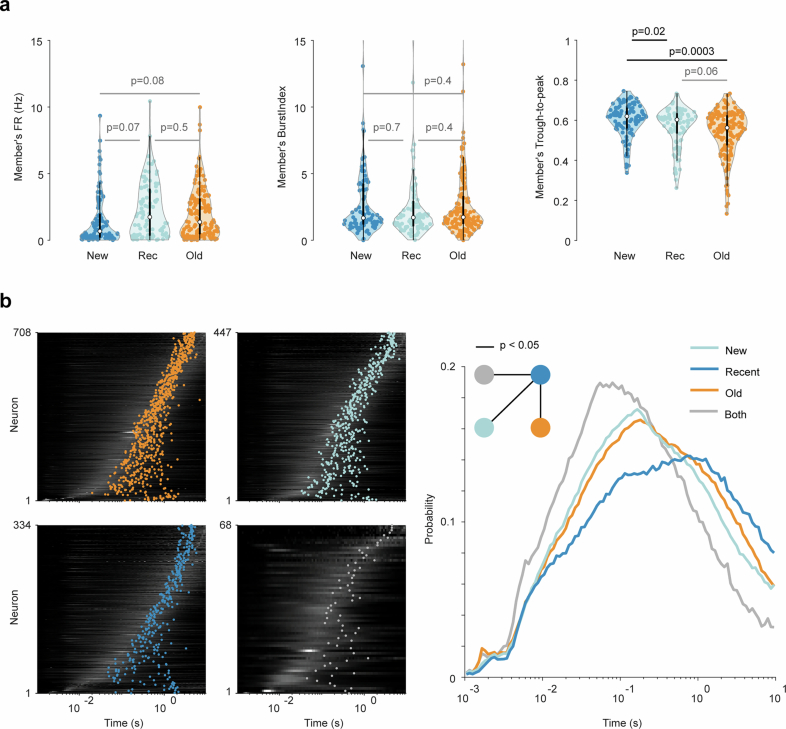
Physiological features of assembly member neurons. **a)** From left to right, distributions of firing rate, burst index, and waveform trough-to-peak duration for neurons belonging to new-, recent-, and old-biased assemblies (n = 94, 92, 147 assemblies for New, Recent and Old, respectively). Statistical tests are based on pairwise differences from two-sided permutation tests with 10000 permutations and FDR correction. The box on violin plots shows the median, and the interquartile range, and the whiskers extend to the most extreme data points within 1.5 × the interquartile range from the lower and upper quartiles. **b)** Left: Interspike interval (ISI) distributions of member neurons in each assembly category, sorted by peak ISI. Colored dots indicate the mean ISI for each group (New: cyan; Recent: blue; Old: orange; Both: gray). Both refers to neurons that were members of both old-biased and new- or recent-biased assemblies. Right: Average ISI distributions for each assembly category. The inset shows pairwise differences in mean ISI across groups (p < 0.01, permutation test with FDR correction).

**Extended Data Fig. 9:**
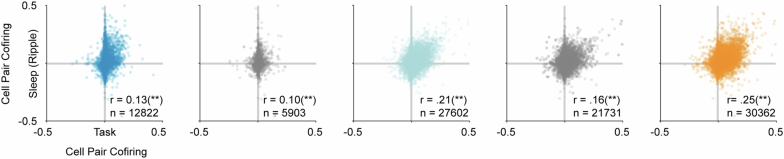
Cofiring dynamics among cell assembly members across brain-behavioral states. Scatterplot of cofiring of pairs of neurons during sleep ripple events as a function of cofiring during task. Cell-pair cofiring was calculated as Spearman’s rank correlation between spike trains for each neuron pair. The consistency of coupling between task and sleep co-firing is quantified with Pearson’s correlation (r).

Assembly and network properties, such as member exclusivity, connectedness, drift and sharp-wave ripple (SWR) modulation, may be attributable to differences in the cell types of the assembly members. We measured four physiological features of assembly members: global firing rate, burst index, waveform trough-to-peak duration and interspike interval (ISI) distributions, for neurons with assemblies that belonged exclusively to new, recent or old assemblies. (Cells with divided memberships were considered separately.) Inhibitory basket cells, for example, are expected to differ from pyramidal cells in their higher firing rates, low bursting activity, shorter (but not burst-interval) ISI and narrow waveshape. We found that firing rates and burst indices did not differ across assembly groups, but the measure of spike width (trough-to-peak slope) indicated narrower spikes for recent and old than for new groups (Extended Data Fig. 8a). Furthermore, mean ISI scores across groups revealed longer ISIs for the new cells than for recent or old cells (Extended Data Fig. 8b; *P* < 0.01; permutation test with FDR correction).

## Superficial and deep CA1 cell participation across assembly type

**Fig. 5:**
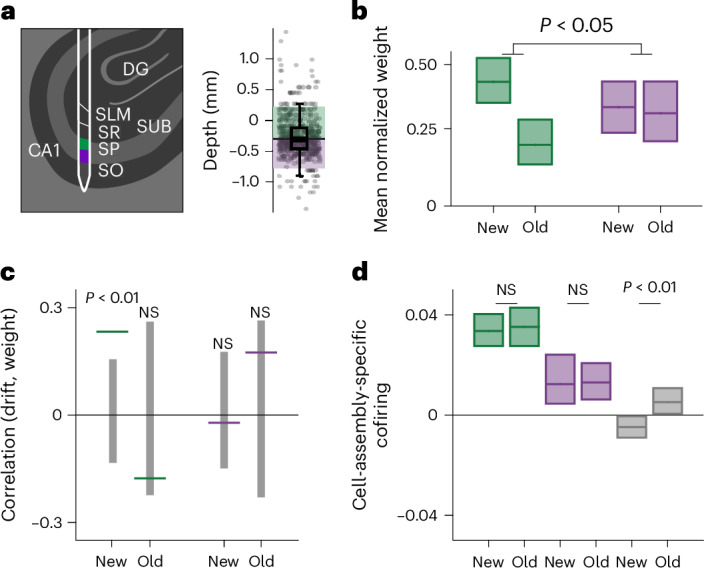
Assembly reorganization by CA1 pyramidal substrata. **a**, Schematic of the laminar profile of one of the deep array electrodes through a coronal view of macaque CA1, highlighting superficial (green) and deep (purple) pyramidal layer substrata. Using methods described previously^47^, including imaging coregistration and functional markers such as ripple source localization, we identified the pyramidal layer across recording sessions and animals. Cells identified as pyramidal based on waveform, ISI and probe location (right, *n* = 631) were subdivided into superficial (green, *n* = 154) and deep (purple, *n* = 147) according to their laminar signal source relative to the pyramidal layer (Methods). The box plot shows the median depth of the identified pyramidal neurons; boxes indicate the interquartile range and the whiskers show the most extreme nonoutlier values. **b**, Contribution of superficial (green) and deep (purple) pyramidal neurons to the formation of new (blue) and old (orange) cell assemblies, quantified as the mean normalized weight in PCA and ICA loadings (see Extended Data Fig. 10, GLM model: ‘Weight ≈ Age × Layer’, with a significant main effect of the age context (new or old), and the age–layer interaction; *P* < 0.05). **c**, Correlation between mean normalized weight and Kendallʼs *τ*, with 95% confidence intervals from a two-sided permutation test (*P* < 0.01). **d**, Cofiring between pairs of superficial (green), deep (purple) and superficial–deep (gray) member neurons within new and old cell assemblies (*P* < 0.01; two-sided permutation test).

**Extended Data Fig. 10:**
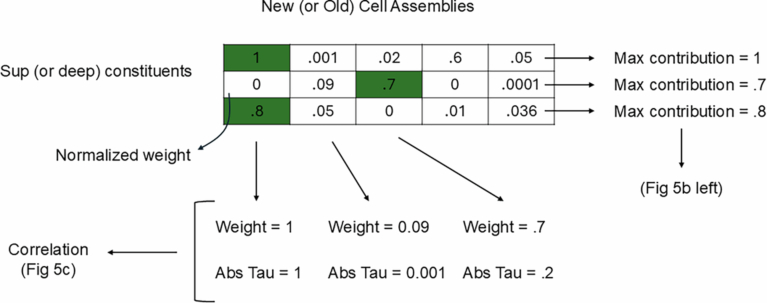
Superficial-Deep Contribution to New and Old Cell Assemblies. (see Materials and Methods for full description).

Monkey CA1 pyramidal cells, like those in rat and mouse CA1, show a laminar segregation of function, including cell assembly membership^47^. In mice, the engagement and functional roles of superficial and deep pyramidal neurons depend on previous experience^48^ and the type of memory being processed^49^. To assess whether a substrata bias exists in monkey assembly participation as a function of experience, we calculated the mean normalized assembly weight of identified superficial and deep CA1 pyramidal neurons, indexing the contribution of each cell to cell assembly dynamics (Fig. 5 and Extended Data Fig. 10). Superficial pyramidal cells preferentially participated in new assemblies, whereas deep pyramidal cells did not (Fig. 5b), similar to observations in rodent CA1^48^. In addition, we found a correlation between the degree of superficial cell participation in an assembly and the future drift rate of that assembly (Fig. 5c), potentially driving the faster drift rates seen in new assemblies. Furthermore, the layer segregation of assemblies was not constant across experience. With assembly age, for those superficial cells remaining, there was greater cofiring with deep cells than that seen for the new assemblies (Fig. 5d). In contrast, intra-sublayer cofiring remained unchanged as a function of age. Together, these findings suggest that superficial pyramidal cells initially contribute more to new assemblies, signaling future drift, and that, over the course of experience, more cross-strata interactions emerge.

## Discussion

This study provides insights into how the primate brain flexibly and dynamically reorganizes as a function of the individual’s history of learned experiences. We recorded from ensembles of single units in freely moving macaques during a memory-guided item-sequence task and in subsequent sleep, revealing the activation and reactivation of hippocampal and extrahippocampal cell assemblies associated with newly, recently and remotely acquired experiences. We found that sleep reactivation is shaped by previous experience, defined by both time and repetition, as older and recently formed memories evoked stronger and more stable assembly reactivation compared to newly learned sequences. In contrast, cell assemblies associated with new experiences underwent flexible reorganization and gradually formed higher-order and temporally extended links with assemblies representing older memories, giving rise to metassemblies—hierarchically coordinated networks that may support the integration of new and old experiences. We found that assemblies composed of more exclusive members were less stable over time (showed greater drift). One possible interpretation of this finding is that the neural ensembles supporting memory favor integration into a broader network, rather than maintaining separability through sparse representations. The potential recruitment of putative inhibitory cell types over time could underlie such a restructuring and stabilization of the network, although this remains to be confirmed in future studies. To further track the cellular basis of these dynamics, we examined the laminar organization of CA1 and found that superficial pyramidal neurons disproportionately contributed to new-biased assemblies and were associated with greater drift, whereas deep pyramidal neurons were more consistent across time. As memories aged, coactivity between superficial and deep pyramidal cells increased, suggesting that, like the metassembly results, subpopulations become progressively intertwined to support established memories. Collectively, these findings highlight differences in the composition, deployment and interactions among memory-related neural ensembles following extended experience involving multiple memoranda. The results additionally implicate network-level features that may underlie long-term memory formation and integration in primates.

Replay—the reinstatement of coordinated cell assembly patterns that were originally expressed during waking experience—is widely regarded as a key systems-level mechanism for memory consolidation, particularly during offline states such as sleep^50–52^. Much of this understanding is based on rodent studies, and evidence for analogous cell assembly dynamics and reactivation processes in primates remains comparatively limited. In monkeys, experience-related reactivation in neural ensembles was first observed during daytime rest and pauses in visual exploration or task performance^8^. In humans, similar reactivation patterns in lateral and medial temporal lobe ensembles have been linked to explicit memory retrieval^10,11,53,54^ and in the human and nonhuman primate motor cortex during postlearning naps^12,55,56^.

Our results extend these findings by demonstrating that, in monkeys, discriminable assemblies observed during extended episodic experiences reactivate during overnight sleep. The assemblies that are presently associated with weeks-old and day-old memoranda reactivated with greater strength during task and sleep than those associated with novel material. Previous studies of reactivation or replay have not compared memoranda that differ in initial experiences with lapses extending over weeks. However, when mazes differ in familiarity, reactivation and replay magnitudes have been reported to favor novelty^57^, familiarity^21,33^ or other factors such as recent repetition and reward^34,58^. By focusing on a cross-sectional rather than longitudinal design, we cannot speak toward changes in the identity of specific neurons comprising the assembly over time. Futhermore, we did not attempt to identify assemblies immediately before the experience. Therefore, we do not make any claims about ‘preplay’ or ‘preconfiguration’ of assemblies. Rather, our claims are focused on systematic differences in the structure and dynamics of assemblies as monkeys use newly learned, recently learned and remotely learned sequential memoranda, as well as on differences in how these assemblies reactivate during ripples in overnight sleep.

To better understand what underlying structure could drive the observed assembly differences, we examined two types of cellular differentiators for assembly membership: CA1 pyramidal substrata and physiological cell type. In superficial CA1 pyramidal cells, we found a greater tendency to participate in new-biased cell assemblies, and their contribution to assembly dynamics predicted the drift rate of the assembly. This finding echoes mouse studies showing greater long-term stability among deep-layer pyramidal neurons, and that superficial neurons exhibit activity changes between novel and familiar environments, whereas deep neurons show no such preference^48^. This may reflect differential synaptic plasticity: in mice, superficial neurons become more potentiated at CA3 synapses following experience, resulting in increased participation and elevated firing rates during post-experience SWRs^59^. In contrast, although in vivo activity depresses CA3 input to deep pyramidal neurons, it does not substantially alter their firing rates across all SWRs. Furthermore, pyramidal cell–interneuron interactions also vary as a function of strata. Interneurons can actively participate in the formation of memory assemblies by modulating their coupling with pyramidal cells during learning. Such interactions persist into sleep^60^, and thus the strata effects may be mediated by their interactions with inhibitory cell populations^47^. Previously, we showed that superficial and deep pyramidal cells exhibit strata-specific spike-timing interactions with inhibitory interneuron groups and tend to form nonoverlapping cell assemblies^47^. In this study, a more detailed analysis revealed that superficial and deep pyramidal cells comprise separate assemblies for new experiences but become more integrated during the processing of established, old memories. In mice, distinct pyramidal subpopulations have been shown to encode different aspects of task structure, such as context, choice and reward configuration^61–63^. Thus, the increased correlation between superficial and deep cells in this study may reflect a strengthening association between contextual cues, correct choices, reward outcomes and affordances embedded in the task environment. A nonmutually exclusive possibility is that the superficial cells are progressively recruited into broader hippocampal cell assemblies that may serve to stabilize memory representations and enhance robustness, although potentially at the cost of increased mnemonic rigidity^49^. A second exploratory analysis evaluated the physiological characteristics of cells used to differentiate cell types. Our previous study identified, within the laminar structure of macaque CA1, multiple candidate inhibitory groups alongside pyramidal cells^47^. In the present study, using all available cells and sites, we found that members of recent and old assemblies had more members with narrower spike widths and shorter ISIs but showed no difference in bursting or firing rate. The more rapid (but nonbursting) intervals might speak toward plasticity or other changes in excitability of the same cells over time. However, this would not account for the increase in narrow spike widths, which is considered a stable cellular characteristic commonly associated with subsets of inhibitory cell classes. An increase in inhibitory cell composition in the recent/old assemblies would also be expected to produce shorter ISIs; however, we failed to find the predicted, concomitant increases in firing rate, potentially as a consequence of differing sensitivity of each method relative to the underlying distribution among cell types. If high-firing-rate inhibitory cells (for example, basket or chandelier cells) are indeed recruited into assemblies during sleep, this could account for the increased connectivity and drift stabilization we observed and could support the higher-order selection of metassemblies. However, we caution that the physiological differences observed will require support from alternative methods designed to track identified cell types during the establishment and maturation of assemblies over time to provide definitive answers to these intriguing possibilities. Together, these findings indicate that cell assembly reorganization may be constrained by CA1 microstructure^47^, and that additional, possibly inhibitory cell types may be recruited, with or without changes in the excitability of existing members.

Assemblies differed not only in their reactivation according to their age categories but also in their interactions with other assemblies. During the task, a given assembly showed a wide range of preferences to coactivate with (or avoid activating with) other assemblies (metassemblies), regardless of whether the pairs were associated with new, old or both memoranda. In sleep, however, these pairwise preferences from the task were preserved only when the metassemblies included the recent or old assemblies. One possible advantage to this higher-order structure is that such interactions support the representation of extended experiences^35,64,65^ and enable the integration of distinct memories separated by time^16,66^. Additionally, according to the ‘information overlap to abstract’ theory, higher-order recombination of memory elements facilitates schema formation by selectively reinforcing shared features across related experiences^67–69^. This mechanism is critical for generating abstract, generalized knowledge^70,71^. Supporting this, empirical findings show that by flexibly linking smaller neuronal subpopulations into longer sequences, the hippocampus can spontaneously generate internal predictive models used in future experiences^72^. Once such a model is established, the memory system can bias replay content toward elements consistent across experiences—effectively sampling from an internalized model of the environment^73^. In this view, existing schemas not only shape how new memories are integrated but also determine which are prioritized for consolidation. Consistent with this, the content of awake SWRs has been proposed to serve as a neurophysiological tagging mechanism to selectively preserve and consolidate aspects of experience for future use^37^. In light of the positioning of SWRs during waking recall of multiple experiences in primates^19,20,74,75^, such a mechanism could extend the role of replay beyond consolidating specific episodes to include a role in extracting and reinforcing generalizable structure.

## Methods

### Subjects and behavioral design

Two adult female macaques (*Macaca mulatta*; 9 years old, referred to as ‘M1’ and ‘M2’) served as subjects in this study. All animal procedures were approved by the Vanderbilt Institutional Animal Care and Use Committee (M1700152) in compliance with the policies of the United States Department of Agriculture and Public Health Service on the humane care and use of laboratory animals. Details of the testing enclosure, task and training were described in a previous study^27^.

#### Trial design

Both monkeys underwent training in a 3D testing enclosure, which allowed them to move freely. This enclosure was equipped with eight touch screens distributed around its periphery (Fig. 1a and Extended Data Fig. 1a). The task started with the presentation of a start cue (black circle) that designated the active screen for the animal. Upon touching the cue and after a 1 s delay, the array onset consisted of the appearance of four objects presented in a 2 × 2 grid on a background scene providing context that was specific for that set of objects. During the selection phase, if the monkeys touched the correct item (‘target’)—the item associated with that screen—a ‘correct’ tone was played as the four objects disappeared. If, instead, they selected one of the three nontarget objects (‘distractors’), an ‘error’ tone was played for 2–4 s, during which the stimuli disappeared, and then the correct target appeared in place. During confirmation, in both cases, before proceeding to the next screen in the sequence (or, after screen 4, reward delivery), the target reappeared at the center of the screen and the monkeys had to touch it. The subjects proceeded through the same trial sequence across all four screens, in order, before receiving fluid reward in the opposite corner of the treehouse. For M1, the total reward was calculated based on the performance of the animal within a trial (for example, two drops of juice for each correct selection and none for incorrect touch) and delivered at the reward receptacle. For M2, a fixed reward amount was delivered, but the reward type (flavor) was switched in the middle of the session to maintain motivation. For the presented data, both subjects always traversed an identical sequence starting with screen 1 (upper left) to screen 4 (lower right) on one corner of the environment.

#### Session design

During each session, the monkeys completed various trial blocks distributed between the new and old conditions. These conditions shared the same trial structure but were situated in opposite corners of the testing apparatus. New trials (day 1) consisted of item-context stimuli that the monkeys had never seen previously and for which they had to learn the associations. The monkeys followed up their training and further learning of the new associations on day 2 (recent trials). Old trials consisted of item-context stimuli that the monkeys had learned 2–9 weeks before testing (presented originally as new sets), with no intervening exposure until recall. For old trials, we concatenated days 1 and 2. Old and new set assignments were balanced across corners, because each new set assigned to the corner opposite its paired old set eventually became an old set itself.

#### Sleep

Following their training session, the monkeys were returned to their housing. For M2, overnight recordings started within approximately 20 min of the training session. For M1, the recordings occasionally included 1–2-h gaps. Sleep epochs occurred with the monkeys in their normal housing area, with their usual social housing accommodations, in complete darkness, following the automated 12/12 h light/dark cycle of the lighting system. Throughout this paper, we used the terms ‘task’ and ‘sleep’ to broadly refer to recordings in the 3D testing enclosure and overnight occupancy in the housing, respectively.

### Electrophysiological recordings

Active multichannel probes were mounted on microdrives and inserted into a chronically implanted base^78^. These included a 128-channel probe (DA128-1; linear configuration with 40-μm contact spacing) for M1 and four 64-channel probes (custom design, organized into four parallel shanks with 40 channels at 90-μm spacing and three shanks with eight channels at 60-μm spacing) for M2 (Deep Array Probes; Diagnostic Biochips; currently distributed by Cambridge NeuroTech). The microdrives (M2: NanoDrives, Cambridge Neurotech; M1: custom, Rogue Research) facilitated precise depth positioning adjustments postimplantation, allowing raising and relowering (maximum: 7 mm and 5 mm, respectively) into target areas while remaining implanted. Postoperatively, the probes were typically advanced in 125-μm steps until the target positions were achieved. For M1, the probe sampled from anterior hippocampal CA1. For M2, two of the probes sampled from anterior CA1, and one sampled the posterior hippocampus, with no verifiable sampling of CA1. In both monkeys, the remaining probe targeted areas in and around the retrosplenial and posteriomedial cortex. Assembly composition by probe is shown in Extended Data Fig. 3. As previously described^47^, the localization of recording sites was verified through postoperative CT scans, coregistered with preoperative MRI data, and also by referencing functional landmarks that changed with increasing depth (for hippocampal probes). Notably, the emergence of depth-specific SWRs within unit-dense layers served as a key reference point for CA1. LFPs were digitally sampled at a rate of 30 kHz using the FreeLynx Wireless Acquisition System (NeuraLynx) and subsequently band-pass filtered between 0.1 Hz and 7,500 Hz. During task performance and sleep recordings for M2, data were wirelessly transmitted to the FreeLynx Acquisition System (NeuraLynx). To optimize battery life, sleep recordings for M1 were stored on an SD card within the acquisition system. A high-frequency noise signal at approximately 6 kHz appeared at low (~10%) battery capacity. The onset of this noise was detected by calculating the root-mean-square envelope of the band-pass-filtered signal between 5,800 Hz and 6,200 Hz. The noise initiation point was defined as the time stamp at which the root-mean-square envelope exceeded 110% of the median value, and sleep data following this point were excluded. For the purpose of isolating stable single units across task and sleep epochs, data were initially converted to microvolt units, bitVolts were standardized to 0.195 and the data were subsequently transformed into binary files encoded as 16-bit integers. Only sessions that exhibited no signal loss during recording were included in the analyses. In LFP-related analyses, the raw signal was subjected to third-order Butterworth filtering with a low-pass cutoff frequency set at either 350 Hz or 450 Hz, and the data were downsampled to 1 kHz. Portions of the data from the CA1-localized probes have been published previously^47^.

### CT–MRI image processing and coregistration

The General Registration tool (Elastix) in Slicer (v.4.11) was used to perform the registration of postoperative CT scans with preoperative MRI images. The registration process maintained default parameters. Preceding registration, the CT images were cropped near to and including the cranium.

### Inertial measurement unit data collection and processing

Inertial data were acquired using a triaxial accelerometer embedded in the FreeLynx system, sampled at 3 kHz along the *x*, *y* and *z* axes. Linear acceleration (BASE_LAX, BASE_LAY and BASE_LAZ) and angular velocity (BASE_AVX, BASE_AVY and BASE_AVZ) signals were collected and *z*-scored. Each signal type was concatenated into a 3D matrix and subjected to PCA to reduce dimensionality and identify the dominant axis of variance. The first principal component from each PCA was extracted and used in subsequent analyses.

### Detecting hippocampal SWRs and estimating ripple slope

A single hippocampal LFP channel with the largest ripple amplitude was selected for ripple detection. The wideband signal was band-pass filtered between 100 Hz and 180 Hz using a third-order Butterworth filter, and squared signal was calculated. The squared signal was further band-pass filtered between 1 Hz and 20 Hz and *z*-normalized. SWR peaks were detected by thresholding the normalized squared signal at 3 s.d. above the mean, and the surrounding SWR start and stop times were identified as crossings of 1 s.d. around this peak. SWR duration limits were set between 40 ms and 400 ms. Adjacent events with an inter-ripple interval of <20 ms were merged.

#### Exclusion criteria

In addition to assessing amplitude and duration, we used several criteria to identify and exclude potential false-positive ripple events.

#### Channel noise exclusion

A ‘noise’ channel, defined as one devoid of detectable SWRs in the LFP, was designated. Any events simultaneously detected on this channel were considered as potential false-positives, likely originating from artifacts such as electromyography^79^.

#### Spectral analysis

We conducted spectral analysis of each detected ripple event to further scrutinize its characteristics. The data were spectrally decomposed using Morlet wavelets, allowing us to compute the frequency spectrum for each event. This was achieved by averaging the normalized instantaneous amplitude within ±50 ms of the ripple peak over the frequency range of 50–200 Hz, normalized by multiplying the amplitude by the frequency. We then analyzed the number and properties of spectral peaks in each detected ripple frequency spectrum. These peaks were identified using the findpeaks function in MATLAB, considering parameters such as peak height, prominence, peak frequency and peak width. This analysis aimed to ensure that the detected ripple events genuinely reflected high-frequency, narrowband bursts within the ripple band range. We applied multiple criteria to achieve this: first, authentic ripple events were expected to exhibit a predominant spectral peak within the ripple band range. Therefore, if no single prominent peak (corresponding to the ripple band) was identified, the event was rejected. Additionally, authentic ripple events were anticipated to display a limited narrowband burst. Thus, if the ripple peak had an excessively wide peak width (indicating broadband spectral changes) or prominent high-frequency activity, the event was considered for rejection^80^.

#### Visual inspection

Finally, all detected ripples underwent visual inspection, and any events flagged as false-positives during this process were subsequently removed from the dataset.

### Spike sorting

Spike sorting was performed using KiloSort 1.0^81^ (https://github.com/cortex-lab/KiloSort). The process involved applying a 300-Hz high-pass filter to the raw signals, followed by whitening the data in blocks of 32 channels. Parameters relevant to automated sorting are detailed in the ‘Kilosort parameters’ section.

#### Removing putative double-counted spikes

The KiloSort algorithm occasionally fits a template to residuals left after another template has been subtracted from the original data, resulting in double-counted spikes. Such double-counted spikes could artificially inflate ISI violations for a single unit or create erroneous zero-time-lag synchrony between neighboring units. Consequently, spikes with peak times within a 5 × 10^−4 ^s interval and peak waveforms detected on the same channels were systematically removed from the dataset.

#### Removing units with artifactual waveforms

KiloSort1 generates templates of a fixed length (2 ms) that match the time course of an extracellularly detected spike waveform. However, there are no constraints on template shape, which means that the algorithm often fits templates to voltage fluctuations with characteristics that could not physically result from the current flow associated with an action potential. Units associated with these templates were considered ‘noise’ and removed on the basis of spread (waveform appears on many channels), shape (for example, no peak and trough or sinusoidal waveform) criteria and autocorrelogram (ACG) function.

#### Manual curation and reclustering with phy

Manual curation and reclustering were performed using phy (https://github.com/kwikteam/phy). KiloSort-derived clusters were imported into phy for manual curation. Units that were poorly isolated according to the initial KiloSort results were reclustered using klusta with custom-designed plugins (https://github.com/petersenpeter/phy2-plugins) to obtain well-isolated single units. The quality of these clusters was evaluated based on refractory period violations and Fisher’s linear discriminant metrics. Noise clusters and poorly isolated units were subsequently excluded from the analysis.

### Kilosort parameters

**Table:** 

ops.datatype	‘dat’
ops.fs	30,000
ops.Nfilt	1,024
ops.whitening	‘full’
ops.nSkipCov	1
ops.whiteningRange	32
ops.criterionNoiseChannels	0.2
ops.Nrank	3
ops.nfullpasses	6
ops.maxFR	20,000
ops.fshigh	300
ops.ntbuff	64
ops.scaleproc	200
ops.NT	32*1024+ ops.ntbuff
ops.Th	[4 10 10]
ops.lam	[10 30 30]
ops.nannealpasses	4
ops.momentum	1./[20 400]
ops.shuffle_clusters	1
ops.mergeT	0.1
ops.splitT	0.1
ops.initialize	‘fromData’
ops.spkTh	−4
ops.loc_range	[3 1]
ops.long_range	[30 6]
ops.maskMaxChannels	5
ops.crit	0.65
ops.nFiltMax	10,000

To remove units with unstable firing rates, we estimated the firing rate of each neuron in 5-min epochs throughout the recording duration. During the task, neurons that ceased firing during any trial block were classified as unstable and excluded from the main analyses. Similarly, during sleep, neurons that stopped firing were also deemed unstable and removed from the primary analyses.

### Classification of deep and superficial CA1 pyramidal cells

The full method was described in a previous study^47^. We developed a semi-supervised method for classifying cell types based on electrophysiological features of recorded units, specifically single-unit waveforms and ISI distributions. Before classification, these features were processed using UMAP to reduce the dimensionality of the feature space. The four UMAP-derived features (two from waveforms and two from ISI) constituted the clustering feature space. Spectral clustering, implemented in MATLAB with Mahalanobis distance and *k* means, was used to identify clusters.

We identified the cluster with units exhibiting low firing rates, high bursting propensity and dense localization within the stratum pyramidale as the pyramidal cell group. These physiological features are hallmarks of CA1 pyramidal cells^82–84^. For superficial and deep analyses, we excluded spatial outliers (units with relative depths outside the 5th–95th percentile range) and cells with mean firing rates >1 Hz or a burst index >3. Because superficial and deep pyramidal cells are classified based on their radial distance within the CA1 pyramidal layer, layer-referenced depth data were required. We used two anterior probes (one per animal) with reliable CA1 sampling across layers.

To estimate linear-array channel locations relative to CA1 layers, we used features of SWRs. The sharp-wave component, generated by CA3 Schaffer collateral inputs, produces a current sink in stratum radiatum and a source in stratum pyramidale, evident as a polarity reversal between these layers. Using current source density analysis of ripple-triggered signals, we identified the stratum radiatum and stratum pyramidale regions. The pyramidal layer was further centered using ripple power and the slope of the sharp-wave envelope peak, with the channel at the slope zero-crossing designated as depth 0.

For each session, cells in the ‘pyramidal’ group (meeting firing rate, burst and depth criteria) were median-split based on depth, creating the superficial group (closer to stratum radiatum) and the deep group (closer to stratum oriens). The spatial distribution of CA1 superficial neurons ranged from −300 μm to 540 μm, whereas CA1 deep neurons spanned −630 μm to −330 μm.

### Burst index

To quantify the burst propensity for each recorded unit, we computed an ACG from the spike times of that unit and derived an index following the approach used in a previous study^85^, operationalized as the ratio of short-latency ACG rate to a long-latency baseline ACG rate. The amplitude of bursting was estimated from the spike ACG (1-ms bin size) as the ratio of the mean ACG rate at short postspike lags (3–5 ms) to the mean ACG rate at longer lags (200–300 ms).

### Decoding of neural population with linear SVM

The decoding analysis followed the guidelines outlined in the STAR protocols^86^. To decode context information from spiking activity, we estimated the firing rates of each neuron during four task periods for each screen performance: (1) approach to screen, (2) cue, (3) selection and (4) confirmation. Each period was assigned one of two context labels: old or new/recent. Peri-reward periods were excluded from the decoding analysis. Firing rates were normalized (*z*-scored) to account for variability across neurons, and neurons with unstable firing during task performance were excluded. For each session, a linear SVM was trained to decode context information (old or new/recent) using either neural population activity (population decoding) or single-unit activity (single-unit decoding). Classification was performed using the ‘fitcsvm’ function in MATLAB. For population decoding, population vectors consisting of firing rates across all included neurons were used as features. For single-unit decoding, the firing rate of an individual neuron served as the feature. Class labels were based on the context (old or new/recent). The linear SVM was chosen for its optimal generalization performance, even with datasets featuring many variables and limited samples, and its robustness to non-Gaussian distributions^87^. Additionally, the linear SVM provides straightforward interpretability, as the activity of each neuron can be directly associated with its weight in the classification task.

The linear SVM has one hyperparameter—the regularizer—also referred to as the *C* parameter. To optimize the performance of the linear SVM, we conducted hyperparameter tuning using a grid search over a range of BoxConstraints values (*C* = [0.001, 0.01, 0.1, 1, 10, 100, 1,000]). The optimal hyperparameters were identified by performing 10-fold cross-validation for each set of hyperparameters. The cross-validation accuracy was calculated as 1 − cvLoss, where cvLoss represents the misclassification rate during cross-validation. We performed hyperparameter tuning for each session. Next, we found the range of *C* parameter that resulted in best performance for the majority of the sessions. For the main SVM decoding, we used this fixed range for all recording sessions.

For the main SVM decoding, we created a design matrix as described. To refine the hyperparameters within the fixed estimated range, we employed a Bayesian optimization approach, in which the BoxConstraints hyperparameter was treated as a continuous variable. The fitcsvm function was used with the ‘OptimizeHyperparameters‘ option, allowing MATLAB’s Bayesian optimization framework to search the hyperparameter space efficiently. The optimization used the expected improvement acquisition function, and the process was capped at a maximum of 100 objective evaluations. After estimating the optimal hyperparameter within the range, we trained a linear SVM model to decode the context from neural population. We used 10-fold cross-validation to estimate the accuracy of the model.

To assess the statistical significance of the decoding accuracy, we performed permutation test with 5,000 permutations. For each permutation, the class labels or firing rates of individual units were shuffled, and the SVM was retrained and tested using the same cross-validation procedure and the hyperparameters of the original model. *P* value was calculated as the proportion of permuted accuracies greater than or equal to the original accuracy. Additionally, bootstrapping (with 10,000 bootstraps) was used to calculate 95% confidence intervals for the permutation accuracies (Extended Data Fig. 2). To account for the number of units included in the decoder and the imbalance in label ratios, which biased both the main and shuffled SVM accuracies (Extended Data Fig. 2), we designed a shuffling test tailored to each session. Statistical significance was then assessed separately within each session.

To assess the effect of heterogeneity within functional subpopulations in which a population is defined by the same sign of the weight, we randomly permuted, without repetition, the vector of firing rates but only across units with the same sign of the weight. The permutation was performed independently on each trial, whereas labels stay untouched (Extended Data Fig. 2).

The weight vector (beta coefficients) of the linear SVM, which represents the contribution of each neuron to the classification decision, was extracted from the final model. The weights were normalized by dividing each weight by the *L*_2_ norm of the entire weight vector to interpret the relative importance of individual neurons in the classification process and for subpopulation analyses described above.

### Gini coefficient

We computed the Gini coefficient, a commonly used measure of sparsity/selectivity, given by

$$G=1-2\frac{\mathop{\sum}\limits_{i=1}^{n}{w}_{i}{a}_{{x}_{i}}}{\mathop{\sum}\limits_{i=1}^{n}|{x}_{i}|}$$

$${w}_{i}=\frac{n-i+0.5}{n}$$

where ${a}_{{x}_{i}}$ is the absolute value of the elements of vector ${\bf{x}}$ sorted in ascending order and n is the number of elements. We used the Typical Sparsity Measures toolbox in MATLAB.

### Assembly pattern identification and activation strength

Cell assemblies were identified during sleep recordings as previously described^30,42,63,88^ (Extended Data Fig. 3). Significant cofiring patterns were detected using an unsupervised statistical method based on ICA. The spike trains for each neuron were binned into time windows of 50 ms (corresponding to the 90th percentile of ripple durations) and *z*-score transformed to eliminate biases attributable to differences in average firing rates. This creates for each session a cell × binned firing rate matrix (*Z*). Next, a PCA was applied to the matrix (*Z*). For this, the correlation matrix of *Z* was given by $C=\,\frac{1}{n}{{ZZ}}^{{\rm{T}}}\,$, and the eigenvalue decomposition of *C* was given by

$$\mathop{\sum }\limits_{j=1}^{n}{\lambda }_{j}{p}_{j}{p}_{j}^{{\rm{T}}}=\frac{1}{n}{{ZZ}}^{{\rm{T}}}$$

where ${\lambda }_{j}$ is the *j*th eigenvalue of *C* and ${p}_{j}$ is its corresponding eigenvector. The Marchenko–Pastur law was used to estimate the number of significant patterns embedded within *Z*. For an *nXB* matrix, an eigenvalue exceeding ${\lambda }_{\max }$, defined by ${\lambda }_{\max }={(1+\sqrt{n/B})}^{2}$, signifies that the pattern given by the corresponding principal component explains more correlation than would be expected if the neurons were independent of each other. The number of eigenvalues exceeding ${\lambda }_{\max }$ was defined as *N*_*A*_ and therefore represents the minimum number of distinct significant patterns in the data. The significant principal components were then projected back onto the binned spike data:

$${Z}_{\mathrm{PROJ}}={P}_{\mathrm{SIGN}}^{{\rm{T}}}Z$$

where ${P}_{\mathrm{SIGN}}$ is the *nXN*_*A*_ matrix, with the *N*_*A*_ principal components as columns.

ICA, using the fast ICA algorithm (http://research.ics.aalto.fi/ica/fastica), was applied to the matrix *Z*_PROJ_. That is, an *N*_*A*_*XN*_*A*_ unmixing matrix *W* was found such that the rows of the matrix $Y={W}^{{\rm{T}}}{Z}_{\mathrm{PROJ}}$ were as independent as possible. The arbitrary signs of the independent component weights were set so that the highest absolute weight was positive. The unmixing matrix *W* was then used to derive the weight of each cell within each assembly ${\bf{V}}={P}_{\mathrm{SIGN}}W$, in which the columns of ${\bf{V}}\,(\mathrm{that}\,\mathrm{is},\,{v}_{1},\,\ldots ,\,{v}_{\mathrm{NA}})$ are the weight vectors of the assembly patterns.

To determine the task activation strength of the assemblies, we tracked each assembly pattern ${v}_{k}$ over time by:

$${A}_{k}\left(t\right)=z{\left(t\right)}^{{\rm{T}}}{P}_{k}z(t)$$

where $z\left(t\right)$ is a smooth vector function containing for each neuron its *z*-scored instantaneous firing rate, and ${P}_{k}$ is the matrix projecting $z\left(t\right)$ to the activation strength of the assembly pattern k at time t.

To estimate the reactivation strength of the assemblies detected during the task, we used task projection matrices as templates. The spiking matrix of the sleep epoch were binned (50 ms), *z*-scored and projected onto the projection matrices, resulting in reaction strengths that track the time-resolved reactivation of target neuronal ensembles relative to the reference epoch. The diagonal of the projection matrices was set to 0 to make the activation and reactivation strengths more robust to the fluctuation in single-neuron activity.

Activation or reactivation vector values greater than 2 times the s.d. of each epoch independently were used as time stamps of significant expression of each assembly during task and sleep, respectively. Activation and reactivation rates were calculated by dividing the number of detected events by the total duration of the epoch of interest.

### Significance test for reactivations

To assess the significance of reactivation events, we performed two separate permutation procedures. No single permutation procedure is able to emulate all possible ways of generating spurious coactivation. Thus, our two approaches were designed to address two different ways of disrupting coactivation with a trade-off between preserving the ISI histograms for each neuron and keeping the simultaneous firing rate modulation intact^1^.

In the first approach, we created surrogate firing rate matrices by shuffling the individual ISIs within the spike train of each neuron. This method preserves ISI histograms for each neuron while compromising the population dynamics and synchronous firing rate fluctuation.

In the second approach, we shuffled the weight of each neuron across the detected templates. In this way, we preserved the ISI histogram of individual neurons and the simultaneous firing of neurons in the firing rate matrix but disrupted the contribution of each neuron to different patterns (exchanging members across cell assemblies). This is a more conservative approach to testing the reactivation events.

We repeated the permutation process for both approaches 5,000 times, and for each permutation, we computed the reactivation vectors based on methods described previously. For each surrogate reactivation vector, we computed the mean of the absolute values and created a surrogate distribution from them. Finally, we compared the mean absolute value of the original reactivation vectors to their corresponding surrogate distributions and estimated the *P* value based on a two-sided test.

### Quality of cell assemblies, assembly membership, sparsity and cosine similarity

Members of candidate cell assemblies were identified using Otsu’s method^32^ to divide the absolute weights into two groups maximizing interclass variance, and neurons in the group with greater absolute weights were classified as members (Extended Data Fig. 3).

Goodness of separation was quantified using OtsuEM, the ratio of the interclass variance to the total variance. Assemblies with small OtsuEM presumably result from limitations of the ICA method in identifying independent components from the principal components^31^. We therefore discarded assemblies with OtsuEM < 0.65 from further analyses.

For each cell assembly weight vector (also referred to as loading or pattern), we computed the Gini coefficients. A high Gini coefficient (close to 1) indicates high sparsity. In the context of cell assemblies, this means that a small subset of neurons is making a large contribution to the activity of the assembly, whereas most neurons have very small or no contribution. Thus, the activity is dominated by a few highly active neurons, making the assembly sparse. A low Gini coefficient (closer to 0) indicates low sparsity (or high uniformity). In this case, most neurons contribute similarly to the cell assembly, indicating that the activity is more evenly distributed and less sparse. There is a relationship between the OtsuEM and Gini coefficients in this context. Because we removed cell assemblies with OtsuEM < 0.65, this affected the lower boundary of the Gini coefficients for all cell assemblies.

To measure the similarity between pairs of cell assembly patterns detected in a session, we normalized each pattern to have unit norm and computed cosine similarity using the following formula:

$$c_s = \frac{\mathbf{A} \cdot \mathbf{B}}{\|\mathbf{A}\| \, \|\mathbf{B}\|}.$$

### Assembly coupling

To track interaction between pairs of cell assemblies across different conditions, we constructed the assembly coupling matrix, defined as the Pearson’s correlation coefficient between the activation and reactivation strength vectors.

### Activation and reactivation participation selectivity

For each session, we created a participation probability matrix, *N* neurons by *A* assemblies, in which each value of the matrix measured how frequently a neuron had participated in the activation and reactivation events of a given cell assembly. Participation in each activation event was defined as having fired at least one spike. The participation probability of a neuron in a specific cell assembly was calculated as the number of activation events in which the neuron fired at least one spike divided by the total number of detected activation events (Extended Data Fig. 7).

If a cell is highly cell-assembly-specific, it would ideally spike with higher proportion within its respective cell assembly and rarely fire during the activation periods of other cell assemblies. To measure the participation selectivity of neurons in cell assembly activation events, we computed the Gini coefficient for each row of the participation matrix. A high Gini coefficient (closer to 1) shows that a cell is highly selective. For each cell assembly, member’s exclusivity was defined as the mean of the participation selectivity across identified members of that cell assembly.

This method is related to spike dispersion and tuning to the preferred cell assembly, as described in a previous study^65^.

### Measuring bias in cell assemblies

Bias in cell assembly activations was assessed using a two-sided binomial test with specified binomial probabilities. For this analysis, we used the MATLAB function ‘myBinomTest‘ (Matthew Nelson, 2024; myBinomTest(s, n, p, Sided), available at https://www.mathworks.com/matlabcentral/fileexchange/24813-mybinomtest-s-n-p-sided, accessed on 2 December 2024).

For each cell assembly, significant activation events during task performance were identified as described and categorized based on their occurrence during old and new (day 1) or recent (day 2) memory processing. Activations during new trials (*s*) were defined as successes. To account for differences in the total time spent in trials of varying memory ages, we adjusted the proposed probability of success (*p*) by weighting it with the fraction of time bins corresponding to new trials.

The myBinomTest function computes the probability (pout) of observing the resulting success ratio (*s*/*n*) or a more extreme value, given a total of *n* outcomes and a success probability of *p*. A cell assembly was classified as old-biased if pout was less than 0.05 and *s*/*n* was less than *p*. Conversely, it was classified as new-biased if pout was less than 0.05 and *s*/*n* was greater than *p*.

### Assembly drift (Kendall’s *τ*)

We used Kendall’s *τ* correlation coefficient to estimate the strength and direction of drift in given cell assembly activation and reactivation (similar to a previous study^39^). We defined drift as the monotonic association between activation or reactivation time stamps and activation or reactivation strength. Kendall’s *τ* is a nonparametric measure that evaluates the correspondence between paired ranks of two variables, providing insight into the ordinal relationship between them. Kendall’s *τ* coefficient ranges between −1 and 1, in which a value of 1 indicates perfect agreement in ranking, −1 indicates perfect disagreement and 0 indicates no association. The corresponding *P* value was computed to assess the statistical significance of the correlation. We estimated Kendall’s *τ* and its significance using the MATLAB function corr, specifying the correlation type as ‘Kendall’. Assemblies with a nonzero slope and *P* < 0.005 after FDR correction for multiple comparisons across all assemblies were indicated as significant.

#### Trend estimation using the Theil–Sen estimator

To quantify temporal trends while minimizing sensitivity to outliers, we used the Theil–Sen estimator, a robust nonparametric regression method. For each time series, slopes were computed for all possible pairs of observations as the change in the dependent variable divided by the change in time. The final slope estimate was defined as the median of these pairwise slopes, yielding a robust estimate of the monotonic trend that does not assume normality of residuals. An intercept was subsequently estimated from the median of the residuals relative to the fitted slope. This approach provides reliable trend estimates in the presence of noise, nonstationarity and occasional extreme values, and is therefore well suited for neural time series data.

### Global and cell-assembly-specific cofiring, connectivity and clustering

For the global measure of cofiring, recordings were segmented into 50-ms time bins, and for each neuron, the number of spikes within each bin was counted and converted to firing rate. Spearman’s rank correlation coefficients were calculated between the firing rate vectors of different neurons, serving as a measure of their cofiring tendencies. We performed this analysis separately for recordings during task and ripple times during sleep.

Based on the global correlation matrix, we created a weighted adjacency matrix for graph theoretical analysis. The values of the adjacency matrix were equal to the correlation matrix if the FDR-corrected *P* value of the correlation was <0.01 and correlation was positive; otherwise, the weighted adjacency value was set to 0. Thus, ${a}_{{ij}}$ and ${w}_{{ij}}$ define whether an edge existed between nodes *i* and j and the strength of that connection.

We computed wCC given by the following formula:

$${c}_{i}^{w}=\frac{1}{{s}_{i}({k}_{i}-1)}\mathop{\sum }\limits_{j,h}\frac{({w}_{{ij}}+{w}_{{ih}})}{2}{a}_{{ij}}{a}_{{ih}}{a}_{{jh}}$$

where ${c}_{i}^{w}$ represents the wCC for node *i*, which quantifies the degree to which node *i* and its neighbors form triangles, considering edge weights. The term ${s}_{i}$ is the sum of the weights of the edges connected to node *i*, defined as${s}_{i}=\,\mathop{\sum}\nolimits_{j}{w}_{{ij}}$. This is the total weight of the edges emanating from node *i*. The degree of node *i*, ${k}_{i}$, corresponds to the number of neighbors of node *i*. The variables ${w}_{{ij}},\,{w}_{{ih}}$ are the weights of the edges connecting node *i* to nodes j and h, respectively. These represent the strength of the relationships or interactions between the nodes. Finally, ${a}_{{ij}}$ denotes the adjacency matrix elements, in which ${a}_{{ij}}=1$ if there is an edge between nodes *i* and j, and 0 otherwise. The product ${a}_{{ij}}{a}_{{ih}}{a}_{{jh}}$ ensures that the formula only counts terms in which *i*, j and h form a closed triangle. To assess systematic differences in the connectedness of assembly members, each assembly contributed the wCCs of its constituent members to the corresponding age group pool (for example, new assembly members’ wCCs were added to the new assembly group for statistical comparison).

We defined a metric, cell-assembly-specific cofiring, to capture the degree to which the structure of correlations among neurons contributing to assembly activation changed across time. For each assembly, we extracted the firing rates for all recorded neurons during each assembly activation event. This provides population vectors for each activation event. We then performed Spearman’s rank correlations among the *z*-scored firing rate of neurons.

As shown in Fig. 4b, to estimate changes in cell-assembly-specific cofiring, we performed the above analysis during early (first 25%) and late (last 25%) activations separately. For each correlation matrix, we computed the mean absolute correlations from the off-diagonal upper part of the correlation matrix. The absolute difference between the early and late mean absolute correlation values provides a measure of the overall change in the correlations.

### Superficial–deep contribution to new and old cell assemblies

To examine the contribution of superficial and deep pyramidal neurons to the formation of new and old cell assemblies, we used physiological and anatomical features, including the burst ISI, waveforms and layer localization, to isolate them from inhibitory cells and assign them to either superficial or deep layer groups (the details were reported previously^47^). We used their estimated weights in PCA and ICA space within biased cell assemblies. Because eigenvector (pattern) weights can vary across sessions as a result of differences in the number of recorded units and the total variance explained by an eigenvector, we developed a robust, session-insensitive measure (Extended Data Fig. 10). We normalized the weights within each pattern by dividing each weight by the maximum absolute weight value.

Because individual neurons are not expected to participate in all assemblies within a category, we identified the maximum normalized weight of superficial or deep pyramidal neurons in new or old cell assemblies during a given session, rather than averaging normalized weights. This value is used in Fig. 5b.

Typically, multiple superficial or deep pyramidal cells are recorded in a session, resulting in estimates of weights for multiple pyramidal cells per cell assembly. However, as specific pyramidal cells are unlikely to contribute to all cell assemblies, we calculated the maximum weight among all recorded superficial or deep pyramidal neurons to determine the maximum contribution of neurons of a given type to a single cell assembly. This single value per cell assembly is correlated with Kendall’s *τ* in Fig. 5c.

### Generalized linear modeling of cell assembly activity

To quantify how cell assembly activations were modulated by behavioral and contextual variables, we fitted GLMs to single-trial assembly activation strength values. For each session, we modeled the activity from each detected assembly separately. The dependent variable was the mean activation strength of each assembly per trial epoch (for example, cue touch, decision or feedback). The following predictors were included in the model: age context (new or old sequence type), screen (ordinal steps 1–4 in sequence), trial epoch (approach, start, array and choice), trial outcome (correct or incorrect) and animal movement metrics (linear velocity and angular velocity). Age, screen, trial epoch and outcome were treated as categorical variables. Interaction terms between age and both screen and trial epoch were included to capture context-specific modulation of activity.

The model formula was specified as follows:

$$\begin{array}{l}{\mathrm{Act}}\,\approx \,{\mathrm{Age}}\,+\,{\mathrm{screen}}\,+\,{\mathrm{trial}}\,{\mathrm{epoch}}\,+\,{\mathrm{outcome}}\,+\,{\mathrm{linear}}\,{\mathrm{velocity}}\\\qquad\quad+\,{\mathrm{angular}}\,{\mathrm{velocity}}\,+\,{\mathrm{age}}:{\mathrm{screen}}\,+\,{\mathrm{age}}:{\mathrm{trial}}\,{\mathrm{epoch}}\end{array}$$

GLMs were fitted using MATLAB’s fitglm function with a normal distribution and identity link function.

### Rastermap

We used Rastermap^77^ to sort units in Fig. 1d,e for illustration purposes only.

### Corner-specific assembly detection and matching

To test whether the observed assembly structure depends on detecting assemblies across the full task space, we repeated the previously described assembly detection procedure independently for spiking activity during task performance in the old and new corners of the environment. For this analysis, only time points during which the animal was actively engaged in the task were included. Spike trains were segmented based on occupancy of the old or new corner, and the full assembly identification pipeline was applied separately to each subset using identical parameters.

To relate corner-specific assemblies to those identified in the original analysis, we quantified the similarity between ICA loading vectors using cosine similarity. For each assembly derived from the full task-space model, we identified the old-corner and new-corner assemblies with the maximum cosine similarity (matched cosine similarity; Extended Data Fig. 4g). We then compared the ICA loadings of assemblies classified as old-biased or new-biased in the original model with the corresponding assemblies obtained from the old-corner-specific and new-corner-specific models.

### Hidden Markov model of assembly activation

To characterize discrete modes of assembly expression over time, we modeled the activation-strength time series x(t) of each assembly with a discrete-emission hidden Markov model (HMM). Before modeling, the reactivation trace of each assembly was temporally smoothed with a Gaussian window (150 ms) to reduce high-frequency noise while preserving state transitions. The smoothed activation vector **x** (length *T*) was required to have nonzero variance and reshaped to a column vector. We then discretized **x** into ${n}_{{\rm{Bins}}}$ equally spaced amplitude bins spanning $\left[\min (x),\max (x)\right]({n}_{\mathrm{Bins}}=100)$, yielding an observed symbol sequence $s\left(t\right)\in \left\{1,\ldots ,{n}_{{\rm{Bins}}}\right\}$ for HMM fitting.

We fitted an HMM with three latent states using MATLAB’s discrete HMM implementation, reflecting low (suppressed), intermediate (baseline) and high (activated) assembly expression regimes. Model parameters were initialized using *k*-means clustering applied to the continuous activation values **x** into three clusters. Cluster labels were ordered by cluster centroid (low→high activation) to impose an interpretable initialization. The initial transition matrix $T{R}_{0}$ was estimated from the *k*-means label sequence by counting state-to-state transitions across consecutive time points and adding a unit pseudocount to all entries, followed by row normalization to avoid zero-probability transitions. The initial emission matrix ${E}_{0}$ was constructed by computing, for each initialized state, the histogram of discretized symbols s(t) observed at time points assigned to that state; unit pseudocounts were added to all symbol counts, and each row was normalized. (States with no assigned samples were initialized with a uniform emission distribution.)

HMM parameters (transition matrix *TR* and emission matrix *E*) were learned from the discretized sequence using expectation–maximization (MATLAB hmmtrain; maximum 2,000 iterations, tolerance 10^−6^). After training, the most likely latent-state sequence was inferred using the Viterbi algorithm (hmmviterbi), yielding a raw state label time series. For interpretability across assemblies and sessions, final state labels were reordered post hoc by the mean continuous activation value within each inferred state such that state 1 corresponded to the lowest activation regime and state 3 to the highest activation regime. The resulting ordered state sequence was used for subsequent analyses of assembly activation dynamics.

### Cross-assembly state transition analysis

To quantify directed interactions between assemblies at the level of discrete activation regimes, we analyzed transitions between the HMM-inferred state sequences of pairs of assemblies within each session. For each assembly, we used the three-state HMM state sequence (state 1: suppressed; state 2: baseline; state 3: activated) derived from the smoothed reactivation traces as described above.

For each session, we computed directed, lagged cross-assembly transition matrices between all ordered pairs of assemblies across and within groups. Given a reference assembly *A* and a target assembly B, we estimated the probability of the target occupying state *j* at time t+Δ conditional on the reference occupying state *i* at time t, with Δ=1 time step (lag = 1):

$$P({S}_{B}(t+\varDelta )=j{\rm{| }}{S}_{A}(t)=i),i,j\in \{1,2,3\}.$$

For each ordered pair, we first computed a 3 × 3 transition count matrix C, where ${C}_{{ij}}$ denotes the number of times ${S}_{A}(t)=i$ and ${S}_{B}(t+\varDelta )=j$ across all valid time points. We then converted counts to a probability matrix P by normalizing counts within each reference state (row-wise normalization). We computed transition matrices for four-pair categories: (1) new→old (reference: new-biased, target: old-biased), (2) old→new, (3) new→new across distinct assemblies and (4) old→old across distinct assemblies. Self-pairs (for example, assembly *k*→assembly *k*) were excluded for within-group comparisons.

### Pairwise cross-correlation of assembly reactivation dynamics

To quantify the temporal coupling between assembly reactivation time courses (bin size of spike matrix = 50 ms), we computed pairwise cross-correlations between assembly reactivation traces. Before correlation analysis, each assembly trace was mean-centered by subtracting its temporal mean to remove baseline offsets. For each pair of assemblies *i* and *j*, we computed the cross-correlation function using MATLAB’s xcorr function (with coefficient normalization ‘coeff’). Cross-correlations were computed for time lags ranging from −*L* to +*L* samples (where *L* = 1,000 ms), yielding a lag vector of length 2L+1. For each unique assembly pair, cross-correlation was computed once and symmetry was exploited by assigning the reversed correlation vector to the opposite matrix entry, ensuring computational efficiency.

### Detecting dynamic changes in neuron–assembly coupling

To identify neurons with contribution to cell assemblies that changed dynamically over time, we quantified the time-resolved relationship between single-neuron spiking activity and assembly reactivation strength during sleep. For each session, spike trains were binned using a boxcar window with a bin size of 50 ms. Assembly templates obtained from the original assembly detection analysis were applied to the binned spike matrices to compute the assembly reactivation strength. To assess how the coupling of individual neurons to assemblies evolved over time, we divided each session into 100 consecutive, nonoverlapping temporal blocks of equal length. Within each block, we computed the Pearson correlation between the binned spike count time series of each neuron and the reactivation strength time series of each assembly. This yielded, for each session, a 3D tensor of neuron–assembly coupling values with dimensions (neurons × assemblies × time blocks).

To summarize temporal trends in neuron–assembly coupling, we quantified the linear change in coupling strength across time blocks for each neuron–assembly pair. For each neuron and assembly, we fitted a linear model relating the correlation coefficient to block index using ordinary least-squares regression (slope of correlation versus time block). The resulting slope parameter provided a measure of whether the association between a neuron and a given assembly increased or decreased over time. These slope matrices were computed to identify example neurons exhibiting dynamic changes in assembly membership. This analysis enabled the identification of neurons with functional coupling to assemblies that strengthened, weakened or reversed, providing evidence for dynamic assembly membership rather than static assignment.

### Statistics and reproducibility

Statistical analyses were performed in MATLAB (R2021; MathWorks) using built-in and custom functions. No statistical method was used to predetermine sample size; sample sizes were chosen on the basis of previous studies in the field and practical considerations related to experimental feasibility. Investigators were not blinded during the experiments or outcome assessment. No animals were excluded from the analyses. Behavioral conditions were counterbalanced whenever possible.

Details of the statistical tests, including test type, test statistics, exact *P* values and sample sizes, are provided in the figure legends, captions and the main text or Methods, as appropriate. Details of the parameters used in computational and statistical models are provided in Methods. *P* values were corrected for multiple comparisons where necessary. For box plots, the center line indicates the median, the box indicates the interquartile range and the whiskers indicate the most extreme nonoutlier values.

Reproducibility was assessed by confirming that the main effects were observed consistently across animals and sessions, as reflected in the reported sample sizes and, where appropriate, the display of individual data points.

### Reporting summary

Further information on research design is available in the Nature Portfolio Reporting Summary linked to this article.

## Online content

Any methods, additional references, Nature Portfolio reporting summaries, source data, extended data, supplementary information, acknowledgements, peer review information; details of author contributions and competing interests; and statements of data and code availability are available at 10.1038/s41593-026-02357-2.

## Supplementary information

Reporting Summary

## Data Availability

Preprocessed data used for generating these results are available via figshare at 10.6084/m9.figshare.31418819 (ref. ^89^).

## Code availability

All custom codes are available via GitHub at https://github.com/hoffman-lab/Manuscripts/tree/main/AbbaspoorAljishiHoffman2025.
