# Supplementary material for: Experience reorganizes content-specific memory traces in macaques
Source: Nat Neurosci. 2026 Jul 29;29(8):1976–86. doi: 10.1038/s41593-026-02357-2 (PMC13433320; doi:10.1038/s41593-026-02357-2)
Supplement: Supplementary file 1 [file 41593_2026_2357_MOESM1_ESM.pdf]

nature portfolio

Corresponding author(s): *Double-anonymous peer review submissions: write DAPR and your manuscript number here instead of author names.*

Last updated by author(s): *YYYY-MM-DD*

# Reporting Summary

Nature Portfolio wishes to improve the reproducibility of the work that we publish. This form provides structure for consistency and transparency in reporting. For further information on Nature Portfolio policies, see our Editorial Policies and the Editorial Policy Checklist.

## Statistics

For all statistical analyses, confirm that the following items are present in the figure legend, table legend, main text, or Methods section.

| n/a | Confirmed | |
|---|---|---|
| ☐ | ☒ | The exact sample size (*n*) for each experimental group/condition, given as a discrete number and unit of measurement |
| ☐ | ☒ | A statement on whether measurements were taken from distinct samples or whether the same sample was measured repeatedly |
| ☐ | ☐ | The statistical test(s) used AND whether they are one- or two-sided<br>*Only common tests should be described solely by name; describe more complex techniques in the Methods section.* |
| ☐ | ☒ | A description of all covariates tested |
| ☐ | ☒ | A description of any assumptions or corrections, such as tests of normality and adjustment for multiple comparisons |
| ☐ | ☒ | A full description of the statistical parameters including central tendency (e.g. means) or other basic estimates (e.g. regression coefficient) AND variation (e.g. standard deviation) or associated estimates of uncertainty (e.g. confidence intervals) |
| ☐ | ☒ | For null hypothesis testing, the test statistic (e.g. *F*, *t*, *r*) with confidence intervals, effect sizes, degrees of freedom and *P* value noted<br>*Give P values as exact values whenever suitable.* |
| ☒ | ☐ | For Bayesian analysis, information on the choice of priors and Markov chain Monte Carlo settings |
| ☐ | ☒ | For hierarchical and complex designs, identification of the appropriate level for tests and full reporting of outcomes |
| ☐ | ☒ | Estimates of effect sizes (e.g. Cohen's *d*, Pearson's *r*), indicating how they were calculated |

*Our web collection on statistics for biologists contains articles on many of the points above.*

## Software and code

Policy information about availability of computer code

| | |
|---|---|
| Data collection | Data analyses were performed using MATLAB (versions 2018 and 2021). All built-in and custom analysis functions are described in the Methods. Custom code used in this study is publicly available at https://github.com/hoffman-lab/Manuscripts/tree/main/AbbaspoorAljishiHoffman2025 |
| Data analysis | The analysis codes are deposited at https://github.com/hoffman-lab/Manuscripts/tree/main/AbbaspoorAljishiHoffman2025.<br><br>We used the following built-in or custom functions in MATLAB:<br><br>- Assembly detection: https://github.com/tortlab/Cell-Assembly-Detection<br>- Measuring Bias in Cell Assemblies: https://www.mathworks.com/matlabcentral/fileexchange/24813-mybinomtest-s-n-p-sided<br>- Hidden Markov Models: kmeans, hmmtrain, hmmviterbi<br>- Support Vector Machine: optimizableVariable, fitcsvm, crossval, kfoldLoss<br>- Generalized Linear Models: fitglm<br>- UMAP: https://www.mathworks.com/matlabcentral/fileexchange/71902-uniform-manifold-approximation-and-projection-umap |

For manuscripts utilizing custom algorithms or software that are central to the research but not yet described in published literature, software must be made available to editors and reviewers. We strongly encourage code deposition in a community repository (e.g. GitHub). See the Nature Portfolio guidelines for submitting code & software for further information.

## Data

Policy information about availability of data

All manuscripts must include a data availability statement. This statement should provide the following information, where applicable:

- Accession codes, unique identifiers, or web links for publicly available datasets
- A description of any restrictions on data availability
- For clinical datasets or third party data, please ensure that the statement adheres to our policy

Data required to reproduce the figures will be deposited on DANDI.

## Research involving human participants, their data, or biological material

Policy information about studies with human participants or human data. See also policy information about sex, gender (identity/presentation), and sexual orientation and race, ethnicity and racism.

| | |
|---|---|
| Reporting on sex and gender | N/A |
| Reporting on race, ethnicity, or other socially relevant groupings | N/A |
| Population characteristics | N/A |
| Recruitment | N/A |
| Ethics oversight | N/A |

Note that full information on the approval of the study protocol must also be provided in the manuscript.

# Field-specific reporting

Please select the one below that is the best fit for your research. If you are not sure, read the appropriate sections before making your selection.

☒ Life sciences ☐ Behavioural & social sciences ☐ Ecological, evolutionary & environmental sciences

For a reference copy of the document with all sections, see nature.com/documents/nr-reporting-summary-flat.pdf

# Life sciences study design

All studies must disclose on these points even when the disclosure is negative.

| | |
|---|---|
| Sample size | Sample sizes determined based on previous n numbers used in the literature for similar experiments. |
| Data exclusions | No animals were excluded from the analyses. Session sleep data following the appearance of a high noise level was excluded. For single-unit analyses, noise clusters and poorly isolated units were excluded. Neurons that ceased firing during any task block were classified as unstable and excluded from the main analyses. For superficial–deep comparisons, spatial outliers were excluded. For SVM population decoding analyses, peri-reward periods were excluded. |
| Replication | Replication of the analysis was achieved by considering different sessions from different animals. |
| Randomization | For behavioral training, old and new corners were counterbalanced. |
| Blinding | Experimenters were not blinded. |

# Reporting for specific materials, systems and methods

We require information from authors about some types of materials, experimental systems and methods used in many studies. Here, indicate whether each material, system or method listed is relevant to your study. If you are not sure if a list item applies to your research, read the appropriate section before selecting a response.

## Materials & experimental systems

| n/a | Involved in the study |
|---|---|
| ☒ | ☐ Antibodies |
| ☒ | ☐ Eukaryotic cell lines |
| ☒ | ☐ Palaeontology and archaeology |
| ☐ | ☒ Animals and other organisms |
| ☒ | ☐ Clinical data |
| ☒ | ☐ Dual use research of concern |
| ☒ | ☐ Plants |

## Methods

| n/a | Involved in the study |
|---|---|
| ☒ | ☐ ChIP-seq |
| ☒ | ☐ Flow cytometry |
| ☒ | ☐ MRI-based neuroimaging |

# Animals and other research organisms

Policy information about studies involving animals; ARRIVE guidelines recommended for reporting animal research, and Sex and Gender in Research

| | |
|---|---|
| Laboratory animals | Rhesus macaque |
| Wild animals | This study did not invlove wild animals. |
| Reporting on sex | Only adult female rhesus macaque were used in this study. |
| Field-collected samples | N/A |
| Ethics oversight | All animal procedures were approved by the Vanderbilt Institutional Animal Care and Use Committee, in compliance with the policies of the United States Department of Agriculture and Public Health Service on the humane care and use of laboratory animals. |

Note that full information on the approval of the study protocol must also be provided in the manuscript.

# Plants

| | |
|---|---|
| Seed stocks | N/A |
| Novel plant genotypes | N/A |
| Authentication | N/A |
